# Multiple Hits on Cerebral Folate, Tetrahydrobiopterin and Dopamine Metabolism in the Pathophysiology of Parkinson’s Disorder: A Limited Study of Post-Mortem Human Brain Tissues

**DOI:** 10.3390/metabo15050307

**Published:** 2025-05-05

**Authors:** Dhruti Balakrishna Doddaballapur, Derren J. Heyes, Jaleel A. Miyan

**Affiliations:** 1 Division of Neuroscience, School of Biological Sciences, Faculty of Biology, Medicine & Health, The University of Manchester, 3.540 Stopford Building, Oxford Road, Manchester M13 9PT, UK; 2 Institute of Biotechnology, Faculty of Science & Engineering, The University of Manchester, 131 Princess Street, Manchester M1 7DN, UK; derren.heyes@manchester.ac.uk

**Keywords:** neurodegeneration, cerebrospinal fluid metabolism, oxidative stress, enzyme dysfunction, choroid plexus, biogenic amine synthesis

## Abstract

**Background**: Parkinson’s disorder (PD) affects around 1:500 individuals and is associated with enlarged ventricles and symptoms of normal pressure hydrocephalus (NPH). These features suggest disrupted cerebrospinal fluid (CSF) dynamics and folate metabolism. With L-DOPA treatment showing diminishing benefits over time, there is an urgent need to investigate upstream metabolic disruptions, including folate and tetrahydrobiopterin (BH4) pathways, in post-mortem CSF and brain tissue to understand their roles in PD pathogenesis. **Methods**: CSF and brain tissue from 20 PD patients (mean age 84 years; 55% male; disease duration 10–30 years) and 20 controls (mean age 82 years; 50% male) were analysed. Western and Dot Blots measured proteins and metabolites, spectroscopic assays assessed enzyme activities, BH4 and Neopterin levels were measured using ELISA, and levels of hydrogen peroxide, used as a proxy for reactive oxygen species, and calcium were quantified using horseradish peroxidase and flame photometry assays, respectively. ClinVar genetic data were analysed for variants in genes encoding key enzymes. Statistical significance was assessed using unpaired *t*-tests (*p* < 0.05). **Results**: All enzymes were significantly reduced in PD compared to controls (*p* < 0.01) except for methyltetrahydrofolate reductase (MTHFR), which was elevated (*p* < 0.0001). Enzymes were functional in control but undetectable in PD CSF except tyrosine hydroxylase (TH). BH4 and Neopterin were elevated in PD CSF (*p* < 0.0001, *p* < 0.001) but significantly reduced (*p* < 0.001) or unchanged in tissue. Peroxide was increased in both PD CSF (*p* < 0.001) and tissue (*p* < 0.0001) selectively inhibiting TH. Calcium was 40% higher in PD than controls (*p* < 0.05). No pathogenic variants in enzyme genes were found in ClinVar data searches, suggesting the observed deficiencies are physiological. **Conclusions**: We identified significant disruptions in folate and BH4 pathways in PD, with enzyme deficiencies, oxidative stress and calcium dysregulation pointing to choroid plexus dysfunction. These findings highlight the choroid plexus and CSF as key players in cerebral metabolism and promote further exploration of these as therapeutic targets to address dopaminergic dysfunction and ventricular enlargement in PD.

## 1. Introduction

Parkinson’s disorder (PD) is a progressive neurodegenerative disorder with a global prevalence of approximately 1 in 500 individuals [1]. Despite its significant personal, societal and healthcare impacts, the precise pathophysiological mechanisms underlying PD remain poorly understood. Current treatments, including dopamine replacement therapies and deep brain stimulation, offer symptomatic relief but fail to address the root causes of the disease or arrest its progression [2,3,4,5]. Emerging evidence suggests a critical role for metabolism occurring in the cerebrospinal fluid (CSF), particularly involving the synthesis of tetrahydrobiopterin (BH4), a critical co-factor in neurotransmitter synthesis, in the development of PD [6,7,8]. BH4 is a vital cofactor in the synthesis of dopamine and other biogenic amine neurotransmitters. BH4 is directly produced from the metabolism of 5 methyl-tetrahydrofolate (vitamin B9), predominantly within the choroid plexus [8,9]. BH4 metabolic changes have previously been reported for Parkinson’s [10]. There is increasing recognition of overlapping clinical and neuropathological features between Parkinson’s disease (PD) and idiopathic normal pressure hydrocephalus (iNPH), including gait disturbances and dopaminergic deficits, suggesting possible shared mechanisms in a subset of patients [11,12,13,14]. Parkinsonism is observed in up to 71% of individuals with iNPH, supporting the relevance of considering these disorders in parallel [14]. These previous findings provide a foundation for exploring the identified but poorly understood mechanisms of PD pathogenesis that are addressed in this study.

### 1.1. BH4 Deficiency in PD

BH4 synthesis relies on the enzymatic activity of GTP cyclohydrolase 1 (GCH1), pyruvoyltetrahydropterin synthase (PTPS) and sepiapterin reductase (SPR) [8,9]. Deficiencies in, or blockade of these enzymes disrupt dopamine synthesis and neurotransmitter balance, contributing to the hallmark motor and non-motor symptoms of PD [15]. Sepiapterin reductase deficiency is clinically known to produce symptoms of dystonia, and speech and motor development issues [16,17]. Moreover, as dopamine is the first of the neurotransmitters produced from tyrosine by tyrosine hydroxylase (TH), there will be additional reduced synthesis of nor-epinephrine, epinephrine and melanin [18]. This produces additional neurological problems as well as being responsible for loss of the melanisation characteristics of the PD substantia nigra. In PD, a pronounced deficiency of SPR shifts BH4 metabolism towards two salvage pathways, which generate reactive oxygen species (ROS) as by-products [9]. This increase in ROS exacerbates oxidative stress, a major factor in the degeneration and loss of dopaminergic neurons. Elevated ROS levels further deplete BH4 by oxidising it to its inactive form, BH2, creating a cycle of oxidative damage and neurotransmitter disruption [11,15,16]. TH is also known to be negatively affected by ROS and oxidative stress, further exacerbating the loss of neurotransmitters.

**Figure 1:**
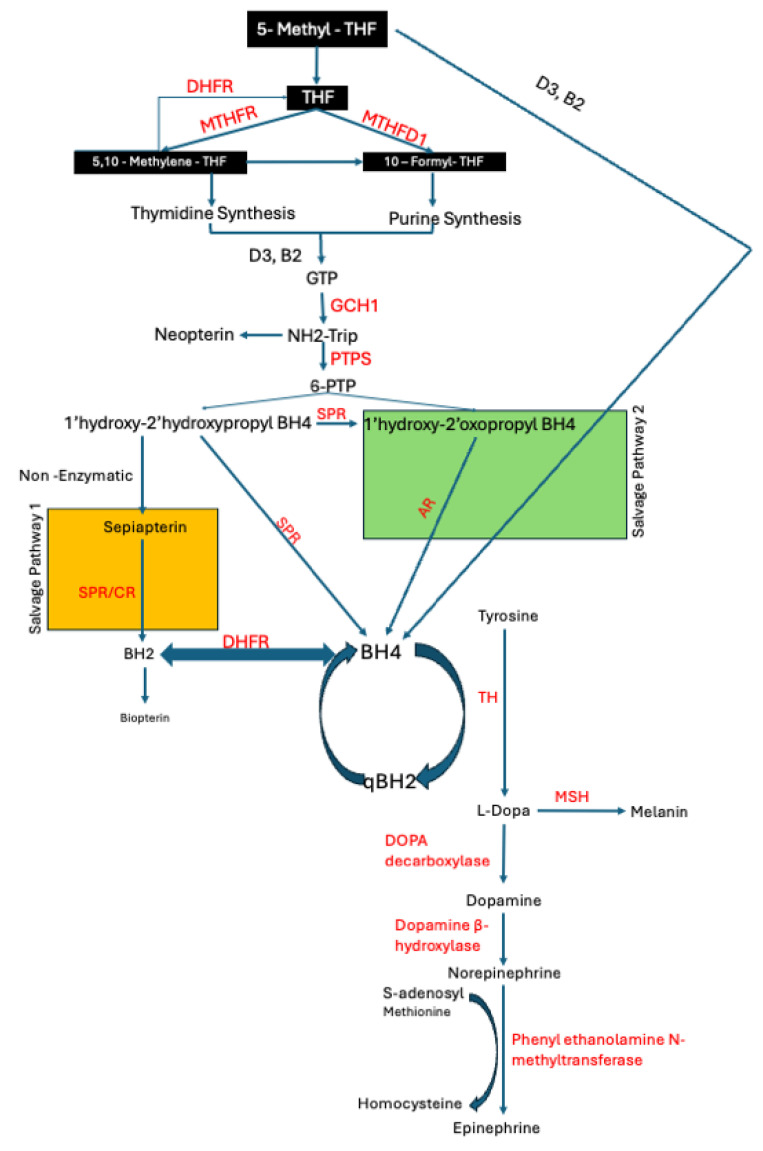
Graphical representation of the pathway involved in neurotransmitter production. Enzymes are shown in red; salvage pathways are depicted in green and orange; black boxes indicate key metabolites of the folate cycle. Arrows indicate direction of enzymatic reactions. THF (tetrahydrofolate), GCHI (GTP cyclohydrolase 1), NH2-Trip (dihydroneopterin triphosphate), SHMT (serine hydroxy methyltransferase), PTPS (pyruvoyltetrahydrobiopterin synthase), SPR (Sepiapterin Reductase), DHFR (dihydrofolate reductase), BH4 (tetrahydrobiopterin), BH2 (Dihydrobiopterin), TH (tyrosine hydroxylase), PH (Phenylalanine hydroxylase), CBR (carboxyl reductase), MSH (Melanocyte Stimulating Hormone).

BH4 synthesis relies on cerebral folate supply and availability. Our previous work, studying cerebral folate metabolism across neuropsychiatric conditions, including psychosis, Schizophrenia, bipolar disorder, PD, epilepsy and multiple sclerosis, suggested the possible involvement of a shared mechanism characterized by insufficient CSF drainage, resulting in ventricular enlargement and subsequent physical and metabolic impacts on the cerebral cortex [19,20]. We found a cerebral folate metabolic blockade similar to that we found in congenital hydrocephalus, indicating that a common outcome of CSF flow or drainage obstruction is a cerebral folate issue [19]. The deficiency of BH4, driven by possible folate deficiency and enzymatic dysfunction, and exacerbated by oxidative stress and iron dysregulation, disrupts the balance of neurotransmitter synthesis in PD. These findings position CSF metabolism and BH4 pathways as central players in the pathogenesis of PD, providing a framework for our current investigations aimed at elucidating upstream metabolic disruptions and their contributions to neurodegeneration in PD. This study aims to explore potential underlying mechanisms, including changes in CSF metabolism and BH4-related pathways, that may contribute to disease progression, with the goal of identifying avenues that may lead to potential therapeutic benefits (refer Figure 1).

### 1.2. Dopamine Synthesis and ROS-Induced Neurodegeneration

Dopamine synthesis begins with the hydroxylation of tyrosine by TH, a reaction requiring BH4 as a cofactor [21]. Age-related and disease-specific increases in oxidative modifications of TH, correlating with reduced enzymatic activity and neuronal viability, have been reported [22,23]. Normally, some amount of L-DOPA is converted into melanin. During a state of oxidative stress, L-DOPA undergoes rapid melanogenesis to increase the protection of Dopaminergic neurons. However, iron deficiency renders L-DOPA unable to convert to melanin, exposing the neurons to degeneration due to oxidative stress [23,24]. A further hit to these neurons is that dopamine stores in the presence of ROS and oxidative stress are acted upon by MAOA, converting them to cytosolic dopamine and causing apoptosis [22].

### 1.3. Iron Dysregulation and Its Contribution to Oxidative Stress

Transferrin bound to iron can interact with transferrin receptors and cross the choroid plexus epithelial cells via receptor-mediated transcytosis into the cerebrospinal fluid (CSF) [25,26]. Additionally, Fe^3^⁺ can dissociate from transferrin within slightly acidic endosomes, be reduced to Fe^2^⁺, and then be transported into the cytosol by divalent metal transporter 1 (DMT1) [25]. In the cytosol, iron is either stored in ferritin or exported into the CSF through ferroportin. Once in the CSF, iron primarily binds to transferrin, which is synthesized or secreted by the choroid plexus [27]. Non-transferrin-bound iron (NTBI) is also present in the CSF, indicating that transferrin in the CSF may be saturated with iron [26].

Iron is critical for various biological processes and is tightly regulated in the brain to prevent toxic accumulation. In PD, disrupted iron homeostasis contributes to oxidative stress via Fenton reactions, leading to the production of ROS that exacerbate neuronal damage [28,29]. Elevated iron levels in the SNpc are consistently observed in PD patients and animal models, correlating with the loss of dopaminergic neurons [27,30]. Iron also influences dopamine metabolism by interacting with tetrahydrobiopterin (BH4)-dependent enzymatic reactions [31]. Excessive iron can oxidize BH4, impairing its function and further disrupting dopamine synthesis [7,25]. This complex interplay between iron dysregulation, BH4 deficiency and ROS generation underscores a multifaceted mechanism of neurodegeneration in PD [9].

### 1.4. Role of Calcium in Lewy Body Formation

Calcium plays a vital role in the brain, acting as a critical signaling molecule in neuronal communication, synaptic plasticity and memory formation [32,33]. It regulates neurotransmitter release at synapses and supports various intracellular processes, including gene expression and metabolic activity [34]. Calcium binding to the C- [35] domain of α-synuclein influences its interaction with synaptic vesicles, playing a pivotal role in synaptic function [35]. This modulation is implicated in PD pathophysiology, highlighting the importance of calcium-mediated α-synuclein dynamics in neuronal dysfunction and offering potential insights for therapeutic interventions targeting synaptic mechanisms [34,36,37].

### 1.5. Aims and Objectives

To better understand the pathophysiology of PD, it is essential to investigate the key elements of the pathways discussed above to identify metabolic faults and, most importantly, any top-level fault that may be responsible for the downstream effects. We used Western blots to identify any changes in concentrations of the key enzymes: MTHFR, MTHFD1, DHFR, GCH1, SPR, PTPS and TH in post-mortem CSF and cerebral cortex tissue of PD patients compared to control samples. GCH1 is involved in the first of three steps of BH4 production, and the absence of this enzyme can be linked to striatal DA depletion and nigrostriatal loss. PTPS is involved in the next step in making BH4. Similarly, SPR is a key enzyme in the last step of BH4 production, with any depletion or blockade leading to the activation of salvage pathways. A deficiency in SPR has been clinically linked to symptoms of dystonia, and speech and motor development issues. Finally, TH is the enzyme responsible for dopamine production and may also be affected. By quantifying these enzymes, we can determine the location(s) of any faults and subsequently identify potential underlying causes. An important aspect of our research was to verify whether the essential reactions, mediated by the enzymes, were proceeding as anticipated or if an unidentified factor was impeding the functioning of the enzymes and the pathway. To investigate this, we performed enzyme assays on specific reactions using both control and PD CSF and tissue samples. These reactions included the production of 5-MTHF, 5,10-methylene THF, THF and L-Dopa. In addition, we measured levels of BH4 and neopterin in CSF and tissue using ELISA kits for accuracy, as BH4 has been found to be reduced with raised neopterin in CSF of PD patients. Neopterin is of interest as a by-product of the pathway to BH4, which has inhibitory effects when raised in concentration. It may therefore have additional negative physiological impact if raised in PD. Our experiments also describe the partitioning of metabolism between CSF and brain tissue.

## 2. Materials and Methods

### 2.1. Sample Collection

Control and Parkinson’s affected CSF was obtained from the Parkinson’s and Multiple Sclerosis Tissue Bank at Imperial College London. All samples were collected after informed consent was provided by the donors or their legal representatives. The Tissue Bank operates under ethical guidelines following the Declaration of Helsinki, and is approved as a Research Tissue Bank by the Wales Research Ethics Committee (Ref. No. 18/WA/0238). As a part of this ethical approval, the Tissue Bank has generic ethical approval on behalf of researchers using tissue or data supplied by the bank. Under conditions agreed with the REC, the Tissue Bank can supply tissue or data to researchers in the UK, without requirement for researchers to apply individually to the REC for approval. All CSF samples were collected from post-mortem patients within 48 h of death and frozen at −80 °C in aliquots of 1 ml. For our experiments we used 20 control samples and 20 Parkinson’s affected samples (details in Supplementary Tables S1 and S2). We also used controls from the Netherlands Brain Bank as a control of controls to normalise the study. An *n* = 40 with 20 controls and 20 PD was used for CSF for this initial study. For tissue, an *n* = 20 was used with 10 control and 10 PD. The tissue was obtained from the pre-frontal cortex and subventricular zones of the brain. All samples were stored in aliquots of 12 μL at −80 °C before use to prevent changes due to repeated freeze and thaw cycles. The age and progression of disease varied among the patients. One control was chosen amongst all the control samples based on neuropathology and western blot results to act as a control of controls, used to normalise all control and affected samples as a reference. This control was obtained from a 70-year-old male in the Netherlands without dementia and presenting with Braak stage II NFT progression linked to age. The sample was collected within 24 h of death and frozen immediately at −80 °C.

Mean age of control samples was 82 years with 50% male, while mean age for PD samples was 84 with 55% males. Duration of disorder ranged from 10–30 years, as highlighted in Table 1. Samples were cross referenced with their comorbidities and medications to account for potential contributions to metabolic changes. Our interpretation of the results is dependent on the accurate reporting of patient comorbidities with special focus on anemia due to its direct link on our metabolic pathways of interest. All samples were analyzed. Outliers were excluded by calculating the Interquartile Range (IQR) and removing samples that deviated significantly from the rest. The experiments were run in triplicate and normalized to a single control to prevent any form of bias.

### 2.2. Western Blots and Dot Blots

Sample aliquots of 6 µL were prepared by diluting in Laemmli buffer (Invitrogen, Glasgow) at a 1:1 ratio with distilled water and 5% mercaptoethanol (mEtOH, Sigma-Aldrich, Poole, UK) to make a total of 12 µL. The mixture was heated at 90 °C for 5 min to denature and reduce proteins to their linear forms for effective electrophoresis. Then, 9 µL of the prepared samples was loaded onto NuPAGETM 4–12% Bis-Tris 12-well precast gels run in NuPAGETM MES running buffer (Thermo Fisher, Paisley, UK) at 120 V for 1 h. Gels were then removed from their plastic casings and transferred onto nitrocellulose membranes using InvitrogenTM Power Blotter Select Transfer stacks (Thermo Fisher, Paisley, UK) using an InvitrogenTM Power Blotter Station at 25 V and 1.3 A for 10 min. For dot blots and total protein assessments, 2 µL of untreated cerebrospinal fluid (CSF) samples were directly applied to dry nitrocellulose membranes and allowed to air dry. Dot blots were made by pipetting 1 ug of CSF onto a piece of nitrocellulose membrane (Amersham Protran 0.45 μm NC Product:10600002) and then drying for 30 min.

Western blot and dot blot membranes were probed with antibodies using the Invitrogen iBind system (Thermo Fisher, Paisley, UK). Membranes were placed face-down on iBind cards pre-soaked in 1× iBind flex buffer. Primary and secondary antibodies were diluted to 2 mL of iBind flex buffer (Supplementary Table S3). Antibodies were sourced from manufacturers with in-house validation to ensure target specificity. Antibodies used in staining have been listed in Supplementary Table S3. The membranes were left in the iBind device until the buffer and antibody solutions were fully depleted or overnight for complete processing. Membranes were scanned using the Bio-Rad ChemiDoc MP imaging system (Bio-Rad Laboratories Ltd., Watford, UK) and image analysis performed with Bio-Rad Image Lab software (version 6.1). Antibody specificity and band positions were verified using blots with positive and negative controls. Positive controls included partial protein antigens provided by the antibody manufacturers, while negative controls omitted the primary antibody. All protein bands were normalized to a standard control included in each gel run. Each sample was analysed at least three times on separate gels for reproducibility. Gel and dot blot images are included in the Supplementary Material, alongside raw and summary data presented in the results.

For tissue, 3 mm^3^ chunks of brain tissue were lysed using PowerBead tubes with ceramic 1.4 mm beads (cat. No. = 13113-50) in 1 ml of Tris-HCl (62.5 mM and pH 6.9) and Sodium dodecyl-sulfate polyacrylamide (SDS) (2% *w*/*v*). Tubes were shaken at high frequency in a Fisher brand Bead Mill 24 homogeniser (Fisher Scientific, Waltham, MA, USA), three times with 3 min intervals of cooling on ice. Following centrifugation, the supernatant was decanted into a clean Eppendorf. Protein concentration was measured using an Implen NanoPhotometer^®^ N60. Sample volumes were adjusted (normalised) to 5 mg/mL protein concentration.

Western blot and Dot blot data was analysed by normalising data to a fixed control. The data was then represented as box and whisker Tukey plots with error bars representing interquartile range (IQR). The statistic test run on this was a MANOVA to understand whether significant differences exist between the individuals, followed by an unpaired T-test between controls and PD affected CSF or tissue for each enzyme. Statistical analysis was carried out using GraphPad Prism (version 9.5.1).

### 2.3. Enzymatic Assays

All the reactions studied involve reduction of a substrate and conversion of NADPH to NADP+. Reactions were measured by following the decrease in fluorescence of NADPH at 340 nm in 96 well UV plates with 97 μL of CSF or tissue lysate in each well. Then, 2 μL substrate and 1 μL NADPH (NADPH, Tetrasodium Salt—Millipore-Sigma, St. Louis, MO, United States) were added and reactions monitored using a Tecan Magellan pro 200 infinite series multiplate reader and Magellan software (version 7.1) Fluorescence scans were set to measure from 340 nm to 480 nm. Data was collected as graphs of Optical density vs. Fluorescence. The rate of reaction was calculated at 460 nm and plotted as box and whisker graphs of control vs. PD. Enzyme activity in both CSF and tissue were measured for the following reactions. Statistical analysis was carried out using GraphPad Prism (version 9.5.1).

ELISA measurements: Tetrahydrobiopterin (BH4) and neopterin (Antibodies.com, Cambridge, UK, catalog number: ABIN6957559) levels in CSF and tissue lysates were quantified using enzyme-linked immunosorbent assay (ELISA) kits. ELISA was performed according to the manufacturer’s instructions using a Tecan Magellan pro 200 multiplate reader and Magellan software. A standard curve was generated for each analyte, and concentrations in the samples were interpolated using linear regression analysis. All measurements were performed in duplicate to ensure accuracy and reproducibility. Statistical analysis was carried out using GraphPad Prism (version 9.5.1), and analyte levels were normalized to protein concentration for tissue samples.

### 2.4. Hydrogen Peroxide Quantification:

The levels of hydrogen peroxide in CSF and tissue homogenates was used as a proxy to measure the amount of ROS produced in the samples and were quantified using a horseradish peroxidase (HRP)-based assay with 2,2′-azino-bis 3-ethylbenzothiazoline-6-sulphonic acid (ABTS, Invitrogen, Glasgow) as the chromogenic substrate. CSF samples were thawed, vortexed briefly, centrifuged at 10,000× *g* for 5 min at 4 °C and the supernatant used in measurements. Tissue samples were homogenized in 50 mM sodium acetate buffer (pH 4.5) containing protease inhibitors using a bead homogenizer, centrifuged at 12,000× *g* for 10 min, and supernatant protein concentration determined using a bicinchoninic acid (BCA) protein assay kit. Working solutions were prepared by combining sodium acetate buffer, hydrogen peroxide (H2O2; final concentration 0.0875 µL/mL) and ABTS (2 mM from a 20 mM stock). For control assays, ascorbic acid (final concentration 0.5 mM) was added. Samples and standards were added to a 96-well plate, followed by the working solution and HRP to initiate the reaction. The reaction catalysed by HRP resulted in the oxidation of ABTS, leading to a colour change to blue, which was measured at 415 nm using a microplate reader (Tecan, Zurich, Switzerland). A standard curve was generated with known hydrogen peroxide concentrations to quantify ROS levels. Tissue ROS levels were normalized to protein content. All experiments were performed in triplicate to ensure reproducibility.

### 2.5. Calcium Quantification

Calcium levels in CSF and tissue homogenates were quantified using flame photometry (Corning Flame Photometer 410). Samples were diluted in deionized water to a final volume of 1 mL for measurements. Calcium standards (ranging from 0.1 mM to 2.0 mM) were prepared using calcium chloride (CaCl_2_, Sigma-Aldrich) in deionized water to generate a calibration curve. A lanthanum chloride (LaCl_3_) solution (0.1% *w*/*v*) was added to all samples and standards at a final concentration of 1% to eliminate potential interference from other ions. The emission intensity at 620 nm, corresponding to calcium, was recorded. A calibration curve was constructed using the standards, and calcium concentrations in the samples were interpolated. All measurements were conducted in duplicate to ensure accuracy and reproducibility. Tissue calcium levels were normalized to protein concentration. Data were analysed using GraphPad Prism (version 9.5.1).

Clinvar Data Analysis: Genetic mutations in SPR, TH, PTPS, MTHFD1, MTHFR and DHFR genes were investigated in the context of Parkinson’s disease (PD) using ClinVar (https://www.ncbi.nlm.nih.gov/clinvar/ accessed on 7 July 2023), a publicly accessible database of clinically relevant genetic variants. Variant data for each gene, particularly those associated with PD, were extracted and assessed for pathogenicity and frequency. Mutations were classified according to ClinVar’s criteria (benign, likely benign, pathogenic, likely pathogenic, and uncertain significance). The distribution of variants in PD patients was compared to controls, aiming to identify potential gene-disease associations and contributing to a better understanding of the genetic underpinnings of PD.

### 2.6. Parkinson’s Patient Involvement Focus Group

A focus group was conducted with 15 participants enrolled in the Patient Involvement Scheme of Parkinson’s UK to explore challenges faced by individuals living with PD. Participants were recruited through Parkinson’s UK, ensuring diverse representation in terms of demographics and disease progression. During the session, participants were presented with an overview of ongoing research into PD. This was followed by a semi-structured discussion where participants shared their personal experiences with PD. To capture comprehensive data, participants completed a survey collecting demographic information (age, ethnicity and family history) and clinical details, including years since diagnosis, medication regimens, diet, underlying conditions, and both reported symptoms of PD (as per NHS guidelines) and unreported changes noticed since diagnosis. Discussions were audio-recorded and transcribed for qualitative thematic analysis, while survey data were analysed quantitatively to identify trends. This mixed-methods approach provided insights into the lived experiences of individuals with PD. Data from this is highlighted in Supplementary Table S4.

## 3. Results

### 3.1. Differences in the Level and Activity of Key Folate and BH4 Enzymes in PD Samples

**Figure 2:**
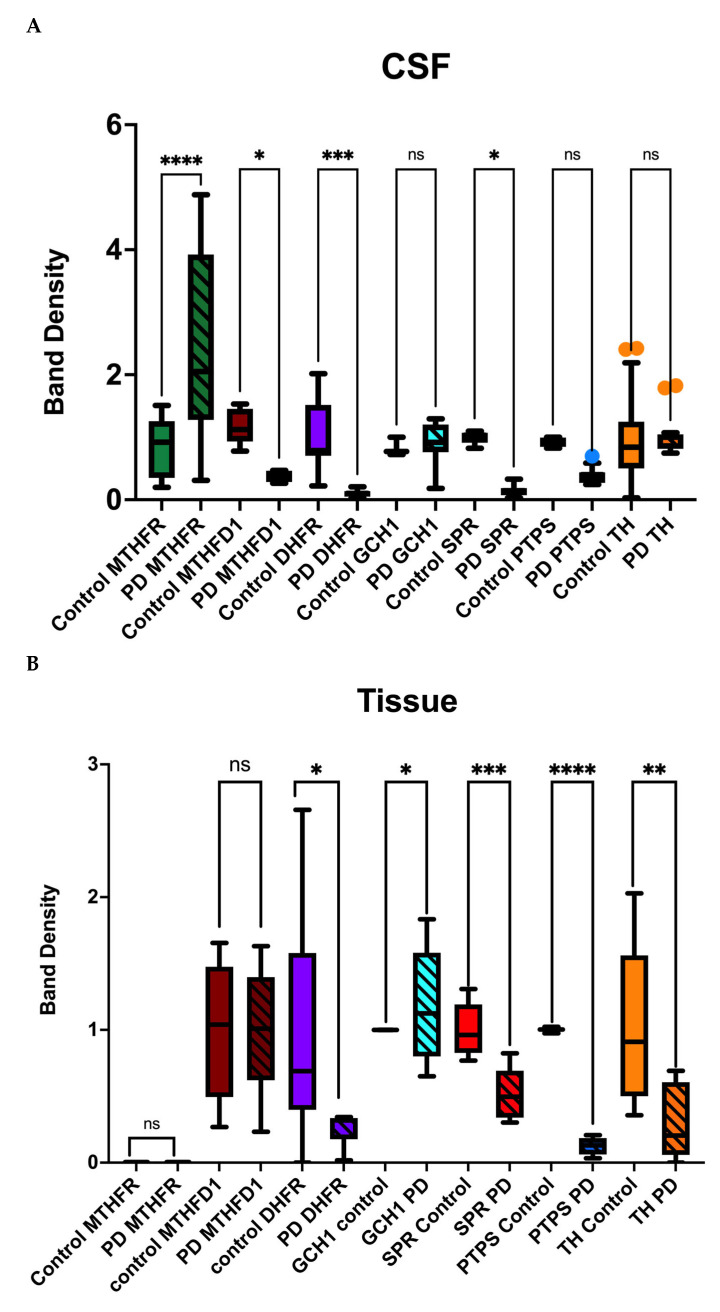

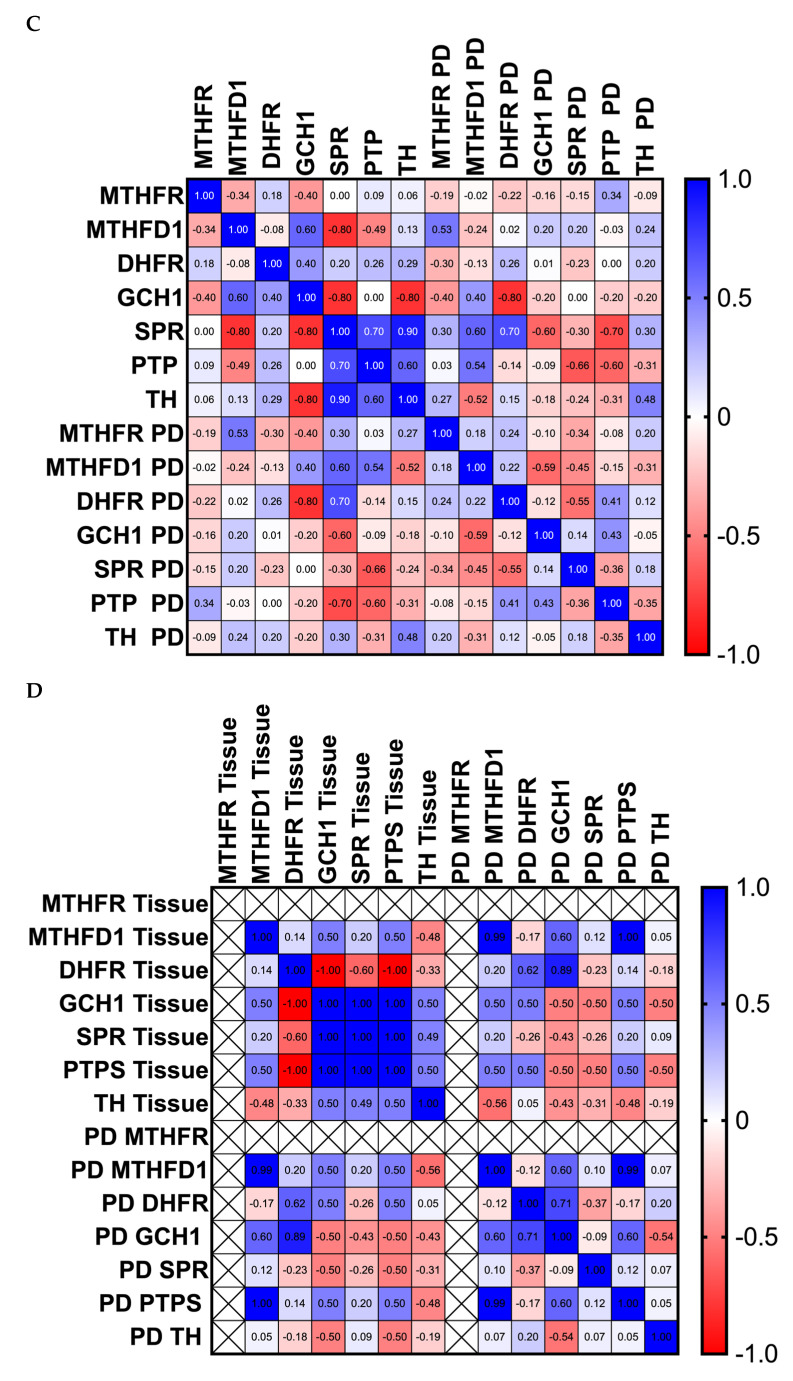
Comparison of changes relative to control in cerebrospinal fluid (CSF) and tissue samples across controls and Parkinson’s disease (PD) groups. (**A**) CSF levels of multiple groups: Controls, PD MTHFR, PD MTHFD1, PD DHFR, GCH1 PD, SPR PD, PTPS PD and TH PD. Data represented as box and whisker plots with error bars representing interquartile range (IQR). Statistically significant differences between groups were analysed using a one-way ANOVA followed by a Šídák’s multiple comparison test. PD MTHFR shows significantly elevated levels (**** *p* < 0.0001), while PD MTHFD1 (** *p* < 0.01), GCH1 PD (* *p* < 0.05), PTPS PD (*** *p* < 0.001) and TH PD (** *p* < 0.01) show smaller but significant changes compared to control. (**B**) Tissue levels of the same groups, comparing disease (PD) and control conditions for MTHFR, MTHFD1, DHFR, GCH1, SPR, PTPS and TH. Significant differences were determined using *t*-tests: GCH1 PD (* *p* < 0.05), SPR PD (* *p* < 0.05), PTPS PD (*** *p* < 0.001) and TH PD (** *p* < 0.01) show significant changes relative to their respective controls, while MTHFD1 shows no significance (ns). (**C**) Correlation Heat Map of enzyme expression in CSF of controls and PD with red indicating negative correlation and blue showing a positive correlation. (**D**) Correlation Heat Map of enzyme expression in tissue of PD and control.

Images of Western and dot blots are provided in Supplementary Figures S1–S26 with density measurements shown in the text. MTHFR, the enzyme recycling folate to methyl-folate, was significantly increased in PD CSF compared to control, while there was no detectable MTHFR in control or PD tissue (Figure 2). MTHFD1, an enzyme involved in 3 critical folate metabolic reactions, was deficient in PD CSF but not significantly different in tissue of PD compared to control. Similarly, DHFR was deficient in PD compared to control in both CSF and tissue. GCH1 was not significantly different in CSF of PD but was significantly higher in PD tissue compared to control. SPR levels in both PD CSF and tissue showed a significant reduction in all samples compared to control. Notably, patients with levels above 0.2 (band density in comparison to control) had been affected with PD for 10 years or less at the time of death while patients with levels below 0.2 had been affected for 20 years or more, indicating a correlation between SPR levels and affected years. From ClinVar genetic data, of 271 suspected PD patients, 220 were diagnosed with Parkinson’s or dystonia [38]. Of these, 90 were identified with likely benign mutations, 76 to have an uncertain significance, 6 were pathogenic, 10 had conflicting consequences, 6 were likely pathogenic and 3 were benign [38]. From this full genome analysis of affected patients, no mutations were found in the SPR gene linked to PD. None of the affected genes had direct relevance to PD pathogenesis and all were related to general energy metabolism, but not in the brain. PTP levels in both PD CSF and tissue were also significantly reduced compared to control (Figure 2).

We found a deficiency in the enzyme 6-pyruvoyl-tetrahydropterin synthase (PTPS), which synthesizes PTP, in PD. This is a known cause of hyperphenylalaninemia due to tetrahydrobiopterin deficiency. BH4 deficiency is also responsible for defective neurotransmission of monoamines because of malfunctioning tyrosine and tryptophan hydroxylases, both BH4-dependent hydroxylase enzymes [9]. The ClinVar data for PTPS identified 52 mutations, but none of the patients affected with these had Parkinson’s or any related disorders, leading to an unlikely relation between PTPS gene mutation and PD pathogenesis [38]. The deficiency is therefore sufficient to cause the problem. TH showed no significant difference in PD CSF compared to the control, but a significant decrease in PD tissue. ClinVar data showed only two gene mutations in TH, but that these had an uncertain association with Parkinson’s pathogenesis [38].

The correlation analysis of enzymes in control CSF shows that MTHFR is negatively correlated with MTHFD1 (*p* = 0.0260) and GCH1 (*p* = 0.075), MTHFD1 is negatively correlated with SPR (*p* = 0.0113) and PTPS (*p* = 0.003) but positively correlated with GCH1 (*p* 0.041) and GCH1 is positively correlated with DHFR (*p* = 0.007). SPR and PTPS are positively correlated with each other (*p* = 0.233) and with TH, respectively (*p* = 0.083 and *p* = 0.242, respectively). However, SPR (*p* = 0.003) and TH (*p* = 0.033) negatively correlated with GCH1, with no correlation between PTPS (*p* = 1) and GCH1 (Figure 2C). Interestingly, when looking at correlation between control and PD for these enzymes, PD either shows a reduced or negative correlation compared to control. In PD, MTHFR shows a positive correlation with MTHFD1 (*p* = 0.0001) and DHFR (*p* = 0.033) and negative correlation with SPR (*p* = 0.05) and PTPS (*p* = 0.007). MTHFD1 shows positive correlation with DHFR (*p* = 0.04) and negative correlation with GCH1 (*p* = 0.049), SPR (*p* = 0.014), PTPS (*p* = 0.006) and TH (*p* = 0.033). DHFR shows a negative correlation with GCH1 (*p* = 0.062) and SPR (*p* = 0.017) but positive correlation with PTPS (*p* = 0.013). GCH1 is positively correlated with SPR (*p* = 0.005) and PTPS (*p* = 0.011) and negatively correlated with TH (*p* = 0.084).

However, in tissue, MTHFD1 (*p* = 0.052) is negatively correlated with GCH1, and SPR is negatively correlated with MTHFD1 (*p* = 0.078) and DHFR (*p* = 0.005). SPR, PTPS and TH are all negatively correlated with each other in PD tissue (*p* = 0.012, *p* = 0.035 and *p* = 0.524, respectively) (Figure 2D).

In PD tissue, MTHFD1 is negatively correlated with DHFR (*p* = 0.774). DHFR is positively correlated with GCH1 (*p* = 0.136) and TH (*p* = 0.613). GCH1 is negatively correlated with SPR (*p* = 0.919) and TH (*p* = 0.297). However, the *p*-values do not indicate a strong significance of the correlation in tissue in PD. This could be because of the small sample size and the variation in disease progression of the samples.

**Figure 3:**
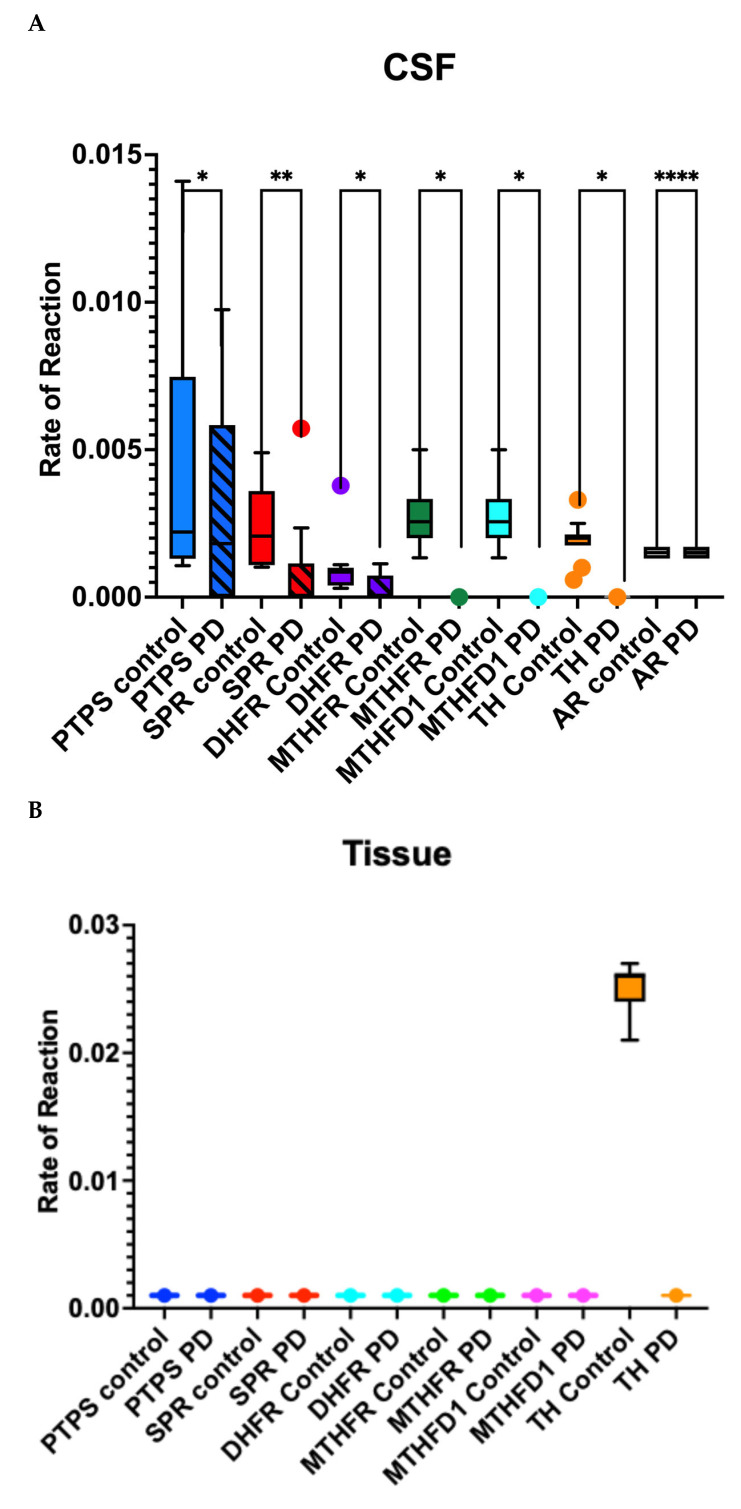

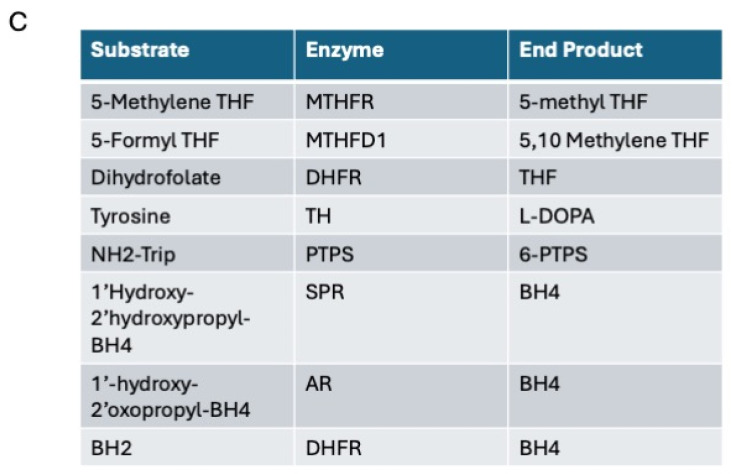
Comparison of the rate of reaction in cerebrospinal fluid (CSF) and tissue samples across controls and Parkinson’s disease (PD) groups. (**A**) CSF rate of reaction measured across various substrates, comparing control and PD groups: PTPS, SPR, DHFR, MTHFR, MTHFD1, TH and AR. Data represented as box and whisker Tukey plots with error bars representing interquartile range (IQR). Statistically significant differences were determined using unpaired *t*-tests. PTPS control (* *p* < 0.05), SPR PD (** *p* < 0.01), DHFR PD (* *p* < 0.05), MTHFD1 PD (* *p* < 0.05), TH PD (* *p* < 0.05) and AR PD (**** *p* < 0.0001) demonstrate significant differences relative to controls. (**B**) Tissue rate of reaction measured in tissue samples for the same substrates (PTPS, SPR, DHFR, MTHFR, MTHFD1 and TH). Statistically significant reductions in the rate of reaction are observed for PTPS (** *p* < 0.01), SPR, DHFR (* *p* < 0.05), MTHFR (** *p* < 0.01) and MTHFD1 (* *p* < 0.05) in PD tissues compared to controls. A significant increase is observed in TH PD relative to controls (**** *p* < 0.0001) (Blots available in Supplementary Figures S1–S26). (**C**) Table showing substrate, enzyme and end products used for enzyme assays. NADPH was added to each reaction as the energy source for the reaction, and the conversion of NADPH to NADP was measured to provide the rates of reactions plotted in (**A**,**B**).

PTPS and SPR activity was only detected in CSF and was depleted in PD CSF compared to control (Figure 3). MTHFR was active in control CSF but not in tissue or in PD CSF or tissue. MTHFD1 showed activity in control CSF but not tissue or in PD CSF or tissue (Figure 3). DHFR was active in control CSF but not in control tissue or PD CSF or tissue (Figure 3). TH activity was limited to control CSF and tissue with no activity of TH in PD (Figure 3). However, the reaction in the salvage pathway II using enzyme AR was active in both control and PD CSF but not in tissue (Figure 3).

**Figure 4:**
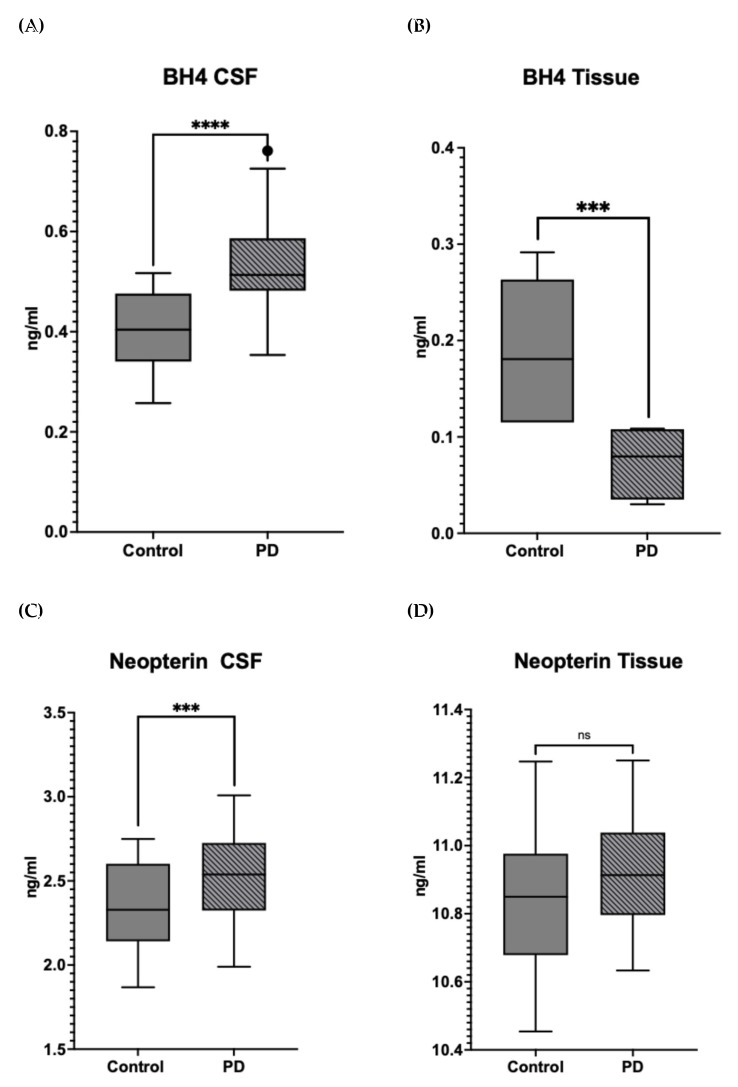
BH4 levels in (**A**) CSF and (**B**) tissue samples from Control and PD groups. BH4 concentration in cerebrospinal fluid (CSF) is significantly increased in the PD group compared to controls (**** *p* < 0.0001). BH4 concentration in tissue is significantly reduced in the PD group compared to controls (*** *p* < 0.001). Data represented as box and whisker Tukey plots with error bars representing interquartile range (IQR). BH4 levels are expressed in ng/mL. Data for concentrations can be found in Supplementary Tables S3 and S4. Neopterin levels in (**C**) CSF and (**D**) tissue samples from Control and PD groups. Neopterin concentration in cerebrospinal fluid (CSF) is significantly increased in the PD group compared to controls (*** *p* < 0.001). Neopterin concentration in tissue shows no significant difference (ns) between the control and PD groups. Data represented as box and whisker Tukey plots with error bars representing interquartile range (IQR). Neopterin levels are expressed in ng/mL. Data for concentrations can be found in Supplementary Tables S5 and S6.

PD patients exhibit altered levels of BH4, neopterin, ROS and calcium in CSF and tissue. We found an increase of BH4 in PD CSF compared to controls but a drop in concentration of BH4 in PD tissue compared to control (Figure 4A,B). Similarly, neopterin was increased in PD CSF as compared to controls but had equal concentration in PD tissue compared to control (Figure 4C,D).

**Figure 5:**
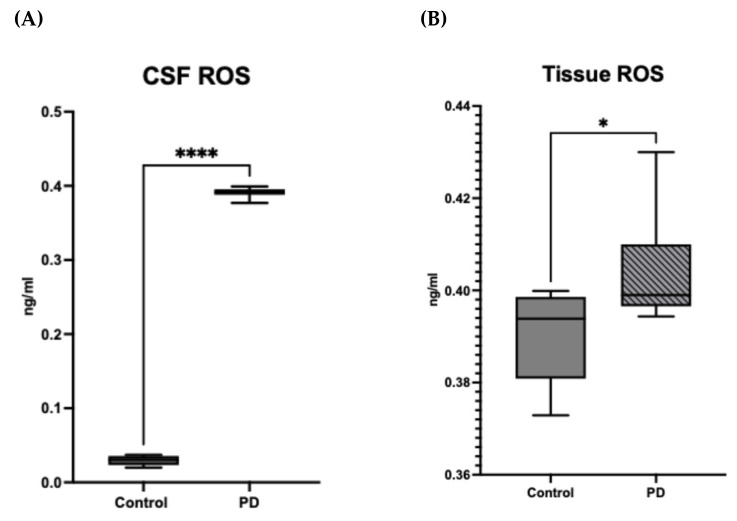

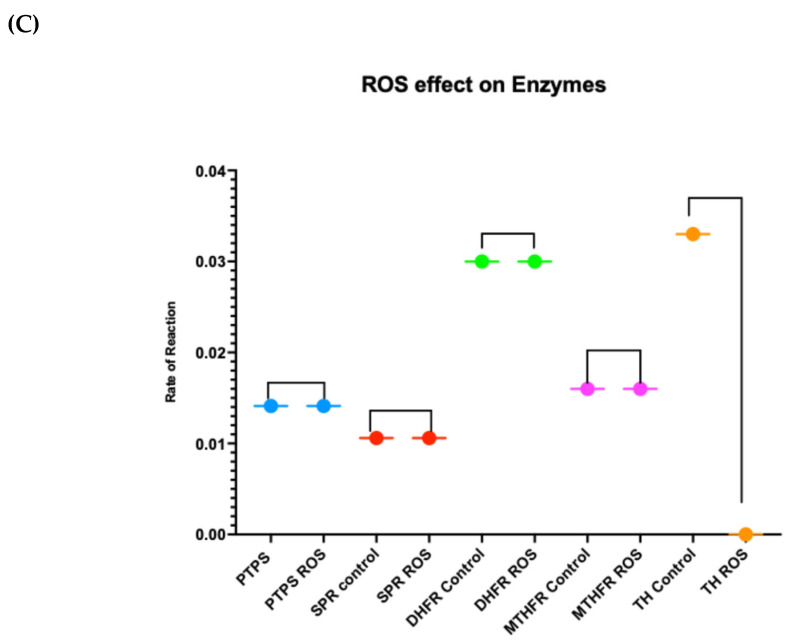
Reactive Oxygen Species (ROS) Levels in (**A**) CSF and (**B**) Tissue Samples from Control and PD groups. ROS concentration in cerebrospinal fluid (CSF) is significantly increased in the PD group compared to controls (* *p* < 0.05). ROS concentration in tissue is significantly elevated in the PD group compared to controls (**** *p* < 0.0001). Data represented as box and whisker Tukey plots with error bars representing interquartile range (IQR). ROS levels are expressed in mg/mL for CSF and ng/mL for tissue showing higher levels of ROS in CSF as compared to tissue. Data for concentrations can be found in Supplementary Tables S7 and S8. (**C**). Effect of Reactive Oxygen Species (ROS) on Enzyme Activity. The box and whiskers plot illustrates the activity levels of six different enzymes under control (solid bars) and ROS-treated (hatched bars) conditions. While most enzymes show no significant change following ROS exposure, tyrosine hydroxylase (TH) is the only enzyme to exhibit a loss of activity under ROS treatment, as indicated by the reduced bar height in the hatched group compared to the control. This suggests that TH is particularly susceptible to oxidative stress, highlighting its sensitivity to ROS.

The HRP assays, which were used to estimate the amount of ROS that had been produced in samples, showed a significant increase in ROS levels in both CSF and tissue of PD compared to controls (Figure 5A). To understand the effects of ROS on enzyme activity in PD, we ran a repeat of the enzyme assays on only control CSF with the addition of hydrogen peroxide (concentration of 35%). This addition did not affect any of the enzymatic reactions except for that of TH, demonstrating specific sensitivity of this enzyme to ROS (Figure 5B).

**Figure 6:**
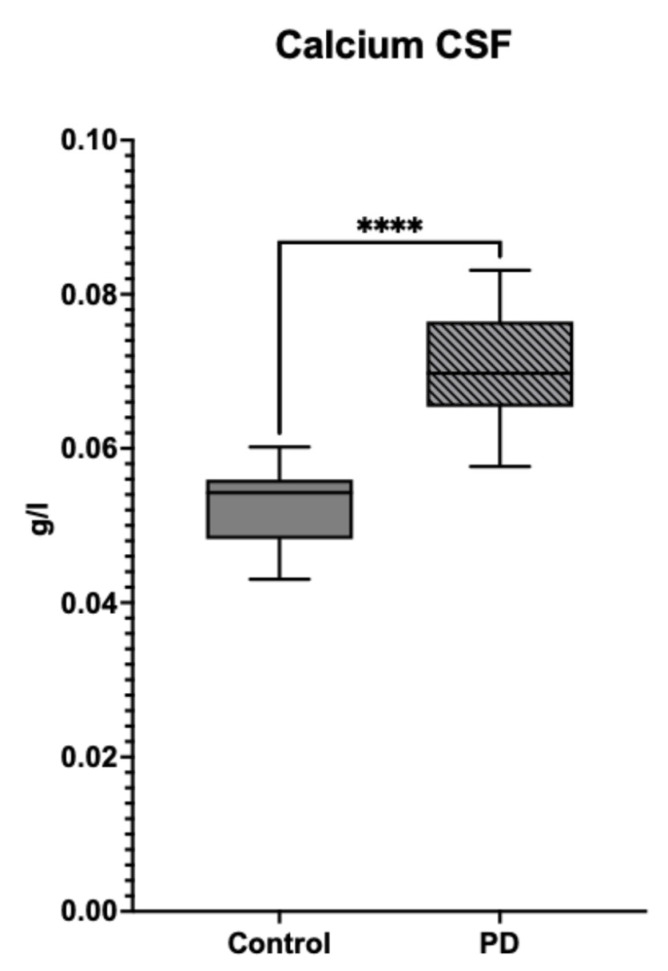
Calcium Levels in Cerebrospinal Fluid (CSF) of Control vs. PD Samples. The bar graph compares the calcium concentrations in cerebrospinal fluid (CSF) between control samples (solid bar) and Parkinson’s disease (PD) samples (hatched bar). The PD group shows a significant increase in calcium levels compared to the control group, as indicated by the greater bar height. Statistical analysis revealed a significant difference between the two groups, with a *p*-value less than 0.0001 (****), indicating that the observed increase in calcium levels is unlikely due to chance. This result suggests a potential association between elevated calcium levels in the CSF and Parkinson’s disease, highlighting the importance of calcium dysregulation in neurodegeneration. Data for concentrations can be found in Supplementary Table S11.

The calcium concentrations were significantly higher in PD CSF compared to control. PD samples showed approximately a 40% increase in calcium levels, suggesting altered calcium regulation in PD (Figure 6). Error bars are present but low, indicating minimal variability in the measurements (Supplementary Table S11).

### 3.2. Effects of Folate Metabolism on PD Patients

Data from the Parkinson’s focus group suggested that PD patients reported that many of their symptoms, developed since diagnosis of the disorder and reducing their quality of life, were directly correlated with identified changes in metabolism (Table 1). Patients that were on folate, vitamin D3, B complex, gut health improving diets and anti-inflammatory supplementation, presented with only a light tremor after 10+ years of being affected by the disorder. However, patients on a purely red meat diet and no supplementation had seen sudden changes for the worse in disease progression over a span of two weeks in the first year of diagnosis.

## 4. Discussion

**Figure 7:**
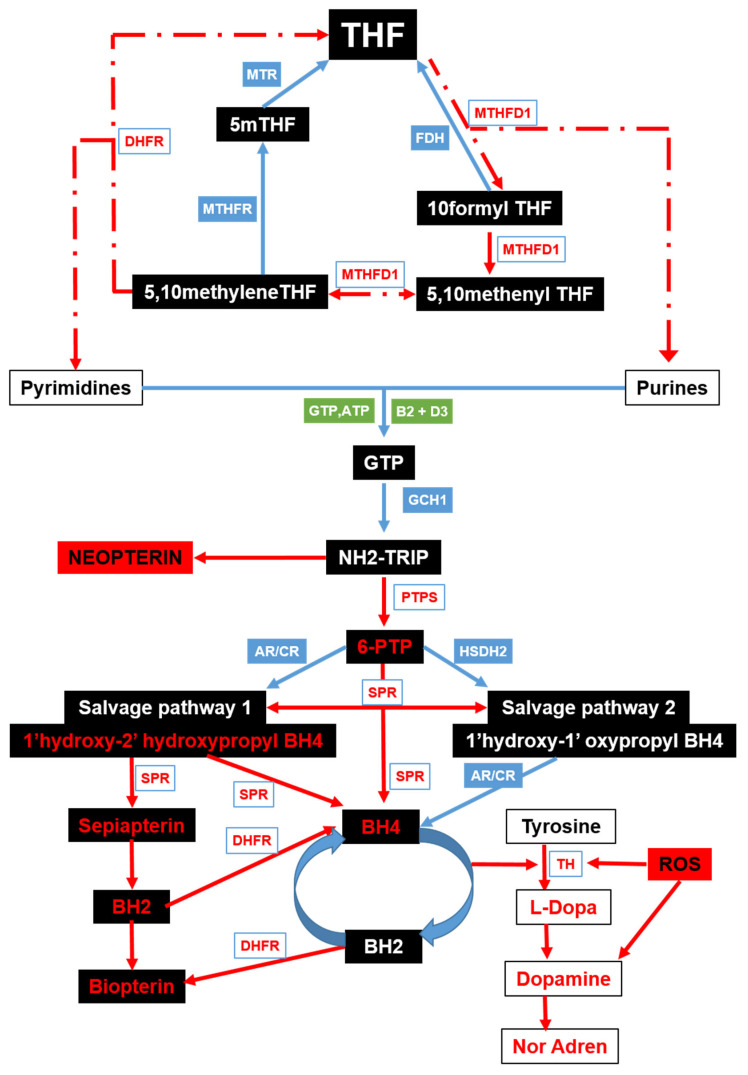
Schematic representation of metabolic faults occurring in PD. Red lines indicate disrupted pathways, dotted lines show enzymatic deficiencies, red enzymes are deficient, blue enzymes are present in normal amounts and blue lines show parts of the pathway working as normal. Increased levels of reactive oxygen species (ROS) and neopterin are indicated in red. Metabolites in red text are deficient due to upstream enzyme faults.

In this study, we explored the potential links between folate metabolism and neurotransmitter synthesis abnormalities and PD. Our analysis revealed significant alterations in the expression and activity of key enzymes involved in these folate pathways in both CSF and tissue samples from PD patients compared to controls. These findings are summarized in the pathway diagram in Figure 7.

MTHFD1 and DHFR were deficient and inactive in both PD CSF and tissue, while MTHFR was overexpressed in PD CSF but was absent in tissue for both control and PD. Importantly, these folate deficiencies directly affect the pyrimidine and purine synthesis pathways and are directly related to anemia due to the error in proliferation of red blood cells [39,40,41]. Reportedly, these changes in synthesis cause loss of psychomotor activity, hypotonia, deafness and visual issues [33,34]. These symptoms can be directly correlated with presentation of PD and interestingly, we observed similar symptoms in PD patients from a PD focus group. The absence of MTHFR and MTHFD1 in tissue but presence in CSF suggests that the choroid plexus and CSF may be sites of synthesis of essential metabolites for neuronal processes [6,11,42,43]. The inactivity of DHFR, MTHFD1 and MTHFR in control tissue but activity in CSF also supports this notion as well as the possibility that DHFR might have a role as a transporter of folate metabolites, similar to FDH, into cells and hence does not show activity in tissue [11]. Our in vitro experiments showed no loss of activity in DHFR, MTHFR or MTHFD1 in the presence of ROS, but account for changes that might occur when the whole system is involved. The lack of MTHFD1 and MTHFR activity in PD CSF could cause deficiency in 5,10 Methylene THF and 5-MTHF, key metabolites in the pathway to BH4 and dopamine. Further downstream of these issues, we observed a marked deficiency in SPR and PTPS levels in both CSF and tissue from PD patients compared to controls, while the expression of GCH1, a rate-limiting enzyme in the BH4 biosynthesis pathway, was similar to the control. By contrast, TH expression appeared normal in CSF but was significantly reduced in tissue. The negative correlation of TH in CSF to tissue can support the hypothesis that TH function is limited to CSF. In addition, SPR and PTPS, which show a positive correlation with each other and TH due to its involvement in BH4 production, are negatively correlated in PD. These findings suggest that SPR and PTPS, key enzymes in the folate cycle, are not functioning optimally in PD, possibly due to impaired enzyme expression or transport, while TH may be hindered in its ability to reach its active form in CSF and tissue.

**Figure 8:**
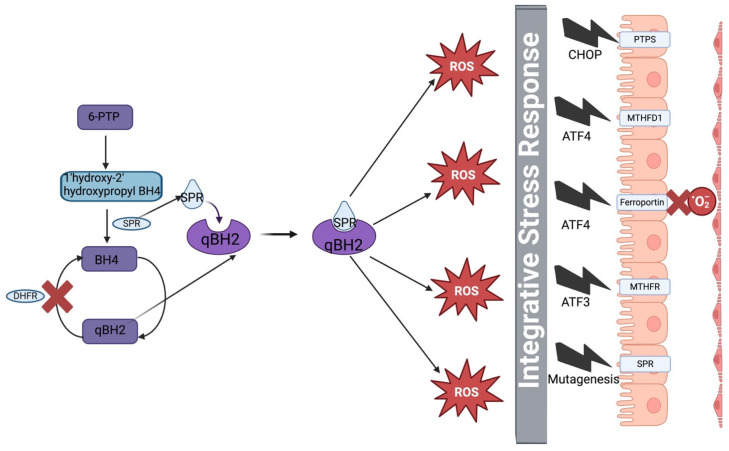
Diagrammatic Representation of Production of ROS in PD and Activation of Integrative Stress Response. The left side of the diagram shows the pathway for BH4 production and its recycling pathway from quinoid BH2 (qBH2) back to BH4 using DHFR. When DHFR is blocked, more qBH2 is produced, leading to SPR binding to it and producing reactive oxygen species (ROS) through the chemical redox cycling pathway. This ROS produces triggers the Integrative Stress Response (IRS) pathway, which releases CHOP, ATF4, ATF3 to block protein synthesis of folate and pterin pathways. Ferroportin is also blocked to prevent oxygen entering the system and forming ROS.

DHFR is sensitive to inhibition by long-term exposure to Zinc, anti-malarial drug induced mutations caused by drug resistance or other environmental or dietary factors. In our pathway, we have seen that DHFR is an important enzyme in the recycling of quinoid BH2 back to BH4 [9,44]. The deficiency of DHFR restricts this recycling pathway, leading to increased levels of qBH2 [9]. This increase in a quinone causes SPR to bind to it immediately, producing cytotoxic reactive oxygen species (ROS) through chemical redox cycling [45]. This increase in ROS triggers the integrative stress response pathway. Integrative stress response (ISR) is a highly conserved biochemical pathway in the choroid plexus that helps it to cope with certain stressors [29,46,47,48,49]. The activation of this pathway triggers the eukaryotic translation initiation factor 2 alpha (eIF2a), which leads to decrease in global protein synthesis, while 43S PIC leads to global attenuation of certain short upstream open reading frame (UORF) enzymes such as ATF4, ATF5, CHOP and GADD34, which aid in cell recovery [29,50]. This then inhibits SPR, blocking it at the C-terminus [45,51]. Interestingly, ATF4, ATF3 and CHOP are responsible in the suppression of protein synthesis of all folate cycle enzymes including MTHFD2 [51,52,53]. This ISR system also downregulates ferroportin receptors to prevent more oxygen coming in, causing production of ROS [25]. This limitation of oxygen, however, puts the system into an environment of hypoxia [31,54]. This limits the availability of oxo-haem groups in CSF [25]. The initiation of the action potential also causes an increase in calcium, which promotes cell death through apoptosis [32,33,35,36,37]. These inhibitions are described in Figure 8.

Tyrosine hydroxylase is dependent on the enzymatic hydroxylation of tyrosine, which occurs when BH4 binds to non-haem iron to form an oxo-iron complex [17,21,31]. The BH4 is oxidized and produces a pterin 4a-carbinoalamine intermediate that can be converted back to BH4 through the recycling pathway [8,9,15,16,17,18,55]. BH4 also acts as a co-factor for nitric oxide synthase, which requires lower amounts of BH4 compared to TH. BH4 acts as a single electron donor to reduce and activate oxygen for the oxidation of NO [55]. The absence or limited availability of BH4 causes the uncoupling of NOS [51], creating a superoxide instead of NO [55]. This is detrimental, as it promotes the oxidation of BH4 to BH2, which is an inactive form known to be competitive with BH4 for NOS binding. This maintains metabolism in a state of BH4 deficiency but completely disrupts the cycle. This deficiency of BH4 in Parkinson’s occurs because of the deficiency in the enzyme Sepiapterin Reductase (SPR), which is necessary for conversion of pterins to BH4.

ROS reduces the availability of free single oxygen atoms, preventing BH4 from being oxidized, which in turn prevents the hydroxylation of tyrosine and dopamine production. The production of ROS, along with the change in pathway and continuous increase in oxidative stress, prevents uptake of enzymes and metabolites into the neurons. Adding BH4 to the enzyme assays did not show any changes in the reactions. However, addition of peroxide to TH produced immediate loss of activity, indicating that the ROS is blocking TH activity. Additional toxicity occurs when any dopamine stores, in the presence of ROS, are acted upon by MAOA to convert it to cytosolic dopamine, which can result in apoptosis of dopaminergic neurons [56,57]. This is a potential mechanism to inactivate TH in presence of ROS to avoid further cell death through buildup of toxic dopamine.

Along with this, PTPS deficiency significantly reduces BH4 production and increases production of neopterin [16]. Neopterin along with ROS, is shown to have toxic effects in silicosis, but its role in the brain is yet to be explored [16,58]. GCH1 is negatively correlated with SPR, PTPS and TH in CSF but positively correlated in tissue. This could be related to production of neopterin. While the amount of neopterin produced in tissue is higher than CSF, there is no change in neopterin levels in PD tissue (Figure 5) but there is an increase in neopterin levels in PD CSF. This negative correlation in control CSF could be an indication of how the increase in GCH1 will increase neopterin production inhibiting downstream activity of PTPS.

The observed deficiencies of MTHFR, MTHFD1, DHFR, SPR and PTPS suggest a direct and critical impact on the choroid plexus in Parkinson’s disease (PD). The occurrence of normal-pressure hydrocephalus (NPH) in PD patients provides additional evidence, as it points to an altered cerebrospinal fluid (CSF) [11,12,13,14,59] composition and impediment to folate metabolism already described in our previous study [19], further implicating the choroid plexus as a potential site of dysfunction. Moreover, recent findings indicate that choroid plexus senescence can drive neuroinflammation by altering its regulatory functions and contributing to immune signaling imbalance [26,27,42,60]. These changes align with the chronic neuroinflammatory environment observed in PD and suggest that the choroid plexus is not only metabolically but also immunologically compromised. These findings collectively position the choroid plexus as a key player in PD pathophysiology [6], warranting deeper investigation into its metabolic and structural changes. The complex nature of changes in metabolism underlying PD exposed in this study indicate that treatments such as L-DOPA may be too simplistic and ineffective in most cases, providing only temporary symptomatic relief at best, as confirmed by our PD patient group.

## 5. Conclusions

Our findings support the hypothesis that Parkinson’s disease may involve a complex metabolic disorder linked to altered CSF composition and enzyme activity, potentially originating from the choroid plexus. This perspective opens new avenues for investigating PD pathophysiology through the lens of CSF dynamics and enzymatic function, but requires further validation through mechanistic studies. To substantiate our hypothesis, further studies will aim to elucidate the mechanisms involved in these changes. Moreover, the observed results will lead to exploring potential epigenetic and nutrigenomic factors that may influence the expression of key enzymes implicated in this disorder. Larger studies of live samples to further validate our data will provide critical insights into the interplay between CSF composition and metabolic regulation, which may lead to potential therapeutic benefits.

Limitations of Study: This study focuses on analysis of post-mortem CSF and tissue, with the potential issues of post-mortem changes. We have tried to negate these changes by using samples with the shortest post-mortem time-to-collection of tissue and a similar time across samples. Further studies will include much larger numbers of samples as well as comparisons to live patient samples. Genetic, epigenetic and nutrigenomic analysis will also be carried out to test for mechanisms of modulation of transcription and/or modification of enzymes, to account for all forms of variability in the data and allow for a data set generalisable to the PD population.

## Figures and Tables

**Table 1:** Data of focus group showing symptoms and related deficiency. Table indicating correlation between symptoms experienced by PD patients in focus group and their underlying deficiencies. Deficiencies and symptoms were provided by the patients involved in the study from private tests.

Symptom	Deficiency
Change in taste	Zinc and vitamin B
Anemia	Folate and B12
Fatigue	Folate/adrenaline
Hair loss	Vitamin D and zinc
Hearing loss	MTHFD1 and MTHFR
Temperature regulation	Low B12, folate and C, changes to ventromedial nucleus of hypothalamus
Clammy skin	Adrenaline
Incontinence	Calcium increase and folate deficiency

## Data Availability

The original contributions presented in this study are included in the article and Supplementary Material. Further inquiries can be directed to the corresponding authors.

## Supplementary Materials

The following supporting information can be downloaded at: https://www.mdpi.com/article/10.3390/metabo15050307/s1, Figure S1: Dot blot image of GCH1 in control CSF, Figure S2: Dot blot image of GCH1 in PD CSF, Figure S3: Dot blot image of GCH1 control tissue, Figure S4: Dot blot image of GCH1 PD tissue, Figure S5: Dot blot image of SPR in control CSF, Figure S6: Dot blot image of SPR in PD CSF, Figure S7: Dot blot image of SPR control tissue, Figure S8: Dot blot image of SPR PD tissue, Figure S9: Western blot image of TH in control CSF, Figure S10: Western blot image of TH in PD CSF, Figure S11: Western blot image of TH control tissue, Figure S12: Western blot image of TH PD tissue, Figure S13: Western blot image of MTHFR in control CSF, Figure S14: Western blot image of MTHFR in PD CSF, Figure S15: Western blot image of MTHFD1 in control CSF, Figure S16: Western blot image of MTHFD1 in PD CSF, Figure S17: Western blot image of MTHFD1 control tissue, Figure S18: Western blot image of MTHFD1 PD tissue, Figure S19: Dot blot image of PTPS in control CSF, Figure S20: Dot blot image of PTPS in PD CSF, Figure S21: Dot blot image of PTPS control tissue, Figure S22: Dot blot image of PTPS in PD Tissue, Figure S23: Dot blot image of DHFR in control CSF, Figure S24: Dot blot image of DHFR in PD CSF, Figure S25: Dot blot image of DHFR control tissue, Figure S26: Dot blot image of DHFR PD tissue, Table S1: Patient details sheet for control samples used for analysis, Table S2: Patient details sheet for PD samples used for analysis, Table S3: List of antibodies, Table S4: Data of focus group showing symptoms and related deficiency, Table S5: BH4 levels (ng/mL) in CSF measured using ELISA, Table S6: BH4 levels (ng/mL) in Tissue using ELISA, Table S7: Neopterin levels (ng/mL) in CSF measured using ELISA, Table S8: Neopterin levels (ng/mL) in Tissue measured using ELISA, Table S9: ROS measured in CSF, Table S10: ROS measured in Tissue, Table S11: Concentrations for Calcium in CSF.

## Abbreviations

The following abbreviations are used in this manuscript:

PD	Parkinson’s Disorder
GCH1	GTP-CYCLOHYDROXYLASE 1
SPR	SEPIAPTERIN REDUCTASE
TH	TYROSINE HYDROXYLASE
MAOA	MONOAMINE OXIDASE A
NOS	NITRIC OXIDE SYNTHASE
ROS	REACTIVE OXYGEN SPECIES
BH4	TETRAHYDROBIPTERIN
MTHFR	METHYL TETRAHYDROFOLATE REDUCTASE
MTHFD1	METHYLENE TETRAHYDROFOLATE DEHYDROGENASE 1
DHFR	DIHYDROFOLATE REDUCTASE
CSF	CEREBROSPINAL FLUID
NADPH	NICOTINAMIDE ADENINE DINUCLEOTIDE PHOSPHATE
PTPS	PYRUVOYL TETRAHYDROPTERIN SYNTASE
CP	CHOROID PLEXUS
SHMT	SERINE HYDROXYMETHYL TRANSFERASE
5-MTHF	5-METHYL TETRAHYDROFOLATE
BH2	DIHYDROBIOPTERIN
AR	ALPHA REDUCTASE
CBR	CARBONYL REDUCTASE
TCA	TRICARBOXYL ACID CYCLE
THF	TETRAHYDROFOLATE
ENT2	Equilibrative Nucleoside Transporter 2
ATP	ADENOSINE TRIPHOSPHATE
