# Supplementary material for: Multiple Hits on Cerebral Folate, Tetrahydrobiopterin and Dopamine Metabolism in the Pathophysiology of Parkinson’s Disorder: A Limited Study of Post-Mortem Human Brain Tissues
Source: Metabolites. 2025 May 5;15(5):307. doi: 10.3390/metabo15050307 (PMC12112997; doi:10.3390/metabo15050307)
Supplement: Supplementary file 1 [file metabolites-15-00307-s001.zip › metabolites-3557302-supplementary.pdf]

Supplementary data

Supplementary Table S1

Patient details sheet for control samples used for analysis.

All samples were obtained from Imperial Brain Bank, London.

Patient ID	Gender	Age at Death	Medication
C087	F	94	NA
PDC034	M	90	NA
PDC035	F	89	NA
PDC059	F	82	Oxycotin
PDC068	F	95	Thyroxine, ISMM, Clopidogrel
PDC084	M	83	Aspirin, Simvastatin, Dipyridamole, Goserelin, Tamsulosin, Dexamethasone, Lansoprazole,
PDC114	M	70	Tramadol 50mg od, Ranitidine 300mg od, Atorvastatin 40mg od, Aspirin 75mg od, Bisoprolol 10mg od, Furosemide 40mg od, Lercanidipine 20mg od, Tamsulosin 400mcg od, Irbesartan 300mg od, Lansoprazole 30mg pd. Insulin: Lantus, Novorapid.
PDC126	M	82	DuoResp Spiromac 160mcg/dose 1-2 puffs twice a day, Spiriva 18mcg one inhalation capsule/day, NovoMix Insulin as required, Paracetamol 500mg-1g prn max qds, Senna 7.5mg two at night, Spirolactone 100mg od, Furosemide 40mg 2 in the morning and 1 at lunchtime, Zopiclone 7.5mg at night, Pregabalin 300mg od, Pregabalin 150mg od, Cholecalciferol 400/Calcium carbonate 1.5g pd, Bisoprolol 5mg od, Simvastatin 20mg at night

PDC128	F	91	Mirtazapine 15mg od, Ranitide 150mg pd, Hydroxychloroquine 200mg pd, Ferrous fumarate 210mg pd, Ascorbic acid 100mg od, Apixaban 2.5mg pd, Alendronic acid 70mg once weekly, Adcal-D3, Loperamide 2mg prn, Paracetamol 500mg 1-2tbl qds/prn.
PDC131	F	92	**Drugs around time of death:** Ranitidine 150mg od, Digoxin 62.5 mcg od, Bisoprolol 2.5mg od, Zopiclone 3.75mg, Pregablin 25mg pd, Cholecalciferol/Calcium carbonate 1.25g pd, Atorvastatin 40mg od, Apixaban 5mg pd,
C05	F	95	**NA**
C08	F	93	**NA**
C013	F	73	**Digoxin**
C030	M	75	**NA**
C075	M	88	**NA**
PDC032	F	91	**NA**
PDC136	F	91	Matrifen patch 25mcg/hour, Cyclizine 50mg tds, Prochlorperazine maleate Buccal 3mg pd
PDC165	NA	NA	**NA**

Supplementary Table S2

Patient details sheet for PD samples used for analysis.

All samples were obtained from Imperial Brain Bank, London. Control sample selected from Netherland Brain Bank samples.

Gender	Age at Death	Years of PD	Symptoms	Other medical conditions	Medication
M	91	11	Tremor in right hand		Rotigotine, Sinemet, Stalevo, Madopar

M	71	22	Memory problems that progressed to dementia, recurrent falls, restlessness at night, peripheral oedema, motor fluctuations, freezing and dyskinesias	Iron deficiency, diastolic heart failure, depression	Sertraline, Omeprazole, Zopiclone
M	87	10	Hypophonia, motor fluctuations, mild paraesthesia (R calf), Dystonia (R leg), late-stage MCI	Hypertension, Trigeminal neuralgia	Sinemet Plus, Sinemet
M	89	12	Dyskinesias, motor fluctuations	IBS, dyspepsia, hypertension, sleep disorder	Mirabegrons, Docusate, Fludrocortisone, Rotigotine, Sinemet
F	82	21	Lewy body dementia	NA	NA
M	82	18	Falls, motor fluctuations	Hypertension	Clopidrogel , Ferrous sulfate , Movicol, Senna, Stanek , Midodrine
F	91	11	Dyskinesia, ansomia, dementia	coronary heart disease, hypothyroidism	Alendronic acid, sinemet plus ,D3 , galantamine , pramipexole
M	71	9	Dysarthria, dysphagia, falls	Obstructive sleep apnoea	rasagiline , ropinirole , co-beneldopa , co-beneldopa , co-beneldopa
M	82	30	NA	NA	NA
F	77	23	Dementia	NA	NA
M	80	11	Cognitive decline and mood changes	NA	Co-careldopa, Diazepam, Mirtazepine , Quetiapine , Zopiclone ,D3 , B12
F	80	23	Frequent falls, insomnia, UTI, bradykinesia, rigidity, festinating gait, weight loss	Depression, dementia, hallucination	Rotigotine 4mg/24 hours, Co-careldopa
F	83	31	NA	NA	NA

M	88	10	Falls, dysphagia	Anaemia	Tramadol 50mg max 8/day, Trimethroprim 200mg pd, Clopidrogel 75mg od, Finasteride 5mg od, Lasoprazole 15mg od, Laxido, Paracetamol 1000mg qds, Rivastigmine 8mg od, Ropinirole 4mg od, Stalevo 150/27.5/200 5/day
M	87	17	Disturbed sleep, freezing and motor fluctuations, mild dyskinesias, Dopamine receptor agonist related confusion	NA	NA
M	NA	10	Falls, motor fluctuations	DVT	Rotigotine 4mg/24h patch
F	83	21	Dyskinesia, falls, motor fluctuations	Fractured left hip	NA
M	NA	25	manual clumsiness, slurred speech, gait difficulty, minor tremor.	Intermittent strabismus	Sinemet, Cabergoline, Pramipexole
F	74	11	Dementia with lewy body	Alzheimer's	Sinemet
M	78	20	NA	NA	NA

Suplementary Table S3

List of antibodies. Table with details on Antibodies Used, Catalogue number, clonality, source (company), host species and working concentration.

Antibody	Vial Number	Clonality	Source	Host species	Working Concentration
MTHFR	29221	Polyclonal	antibodies.com	Goat	1:500
PTPS	110549	Polyclonal	GeneTex	Rabbit	1:1000
MAOA	00047300	Polyclonal	Proteintech	Rabbit	1:500
MTHFD1	10017178	Monoclonal	Proteintech	Mouse	1:1000
TH	00116330	Polyclonal	Proteintech	Rabbit	1:2000
GCH1	00088065	Polyclonal	Proteintech	Rabbit	1:1000
DHFR	00006690	Polyclonal	Proteintech	Rabbit	1:1000
SPR	00008299	Polyclonal	Proteintech	Rabbit	1:500

Secondary Antibody					
StarBright Blue 700 Goat anti-Rabbit IgG	12004162	Polyclonal	BIORAD	Goat	1:4000
StarBright Blue 700 Goat anti-Mouse IgG	12004158	Polyclonal	BIORAD	Goat	1:4000

Spplementary Table S4

Data of focus group showing symptoms and related deficiency. Table indicating correlation between symptoms experienced by PD patients in focus group and their underlying deficiencies. Deficiencies and symptoms were provided by the patients involved in the study from private tests.

Symptom	Deficiency
**Change in taste**	**Zinc and vitamin B**
**Anemia**	**Folate and B12**
**Fatigue**	**Folate/adrenaline**
**Hair loss**	**Vitamin D and zinc**
**Hearing loss**	**MTHFD1 and MTHFR**
**Temperature regulation**	**Low b12, folate and C, changes to ventromedial nucleus of hypothalamus**
**Clammy skin**	**Adrenaline**
**Incontinence**	**Calcium increase and folate deficiency**

Supplementary Table S5

BH4 levels (ng/ml) in CSF measured using ELISA

control	PD
0.2573	0.4916
0.3344	0.5028
0.517	0.5928
0.4041	0.5802
0.4056	0.6839
0.3706	0.3948
0.3259	0.5744
0.4313	0.5134
0.4644	0.5063
0.4783	0.7255

0.3972	0.5408
0.3404	0.472
0.4762	0.7611
0.4884	0.5407
0.4989	0.5746
0.3972	0.6239
0.3813	0.393
0.2683	0.4951
0.4441	0.3535
	0.437
	0.4988

Supplementary Table S6

BH4 levels (ng/ml) in Tissue using ELISA

Control	PD
0.11508	0.030086
0.15365	0.077558
0.291522	0.079818
0.207793	0.035014
0.253935	0.108604
0.11508	0.106
	0.108

Supplementary Table S7

Neopterin levels (ng/ml) in CSF measured using ELISA

Control	PD
2.136786	2.859617
2.33247	3.008208
1.889311	2.564917
2.242857	2.401143
2.647439	2.604001
2.430526	2.757697
1.867549	2.835255
2.400333	2.629246
1.982008	1.990035
2.68087	2.510938
2.154892	2.297929
2.640508	2.434848
2.485374	2.587369

2.320567	2.261118
2.749309	2.101184
2.324462	2.461615

Supplementary Table S8

Neopterin levels (ng/ml) in Tissue measured using ELISA

control	PD
10.78667	10.63345
10.4762	10.93858
11.23004	10.79622
10.64612	11.24905
10.54936	11.08876
10.82072	10.8453
10.93288	10.78791
10.45424	11.00837
10.85721	11.0193
10.94635	11.03857
10.84267	11.01708
10.98627	10.85721
11.24723	10.74319
10.93841	10.91348
10.99193	10.8684
10.7757	10.90827
	10.72232
	11.05641
	11.25037

Supplementary Table S9

ROS measured in CSF

Control	PD
0.037	0.390133
0.02	0.384316
0.036	0.392313
0.037	0.37704
0.025	0.378138
0.029	0.388477
0.023	0.392575
0.027	0.393744
0.033	0.387886
0.032	0.389369

0.035	0.390196
0.02	0.395429
	0.398941
	0.39931
	0.399235
	0.395306

Supplementary Table S10

ROS measured in Tissue

control	PD
0.397314	0.397
0.386489	0.39841
0.399823	0.4
0.399887	0.396095
0.392073	0.399
0.375225	0.394352
0.397013	0.42
0.372906	0.43
0.393861	0.399

Supplementary Table S11

Concentrations for Calcium in CSF

Control	PD
0.054252	0.069328
0.053399	0.057647
0.054252	0.068504
0.044778	0.079106
0.055953	0.08312
0.048247	0.075057
0.043033	0.069328
0.055953	0.063525
0.045648	0.079106
0.049971	0.073428
0.058491	0.078299
0.058491	0.06269
0.060176	0.070151
0.054252	0.069328
0.052545	0.066021
	0.070151
	0.07587
	0.063525

Supplementary Figures: Images of western blots and dot blots

Legend: C stands for control of controls Netherlands sample. The remaining numbers coincide with the patients identification numbers provided in Supplementary Table S1 and Table S2.

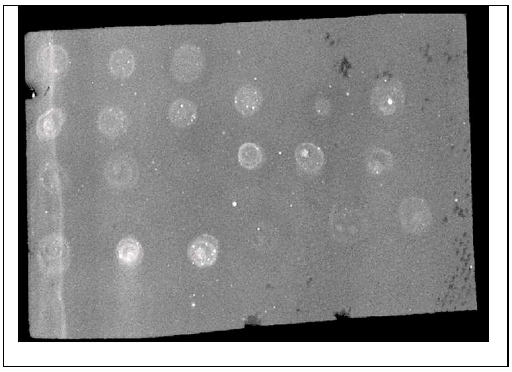

Supplementary Figure S1: Dot blot image of GCH1 in control CSF

C	PDC114	PDC126			
PDC131	PDC68	PDC128	C34	PDC165	C074
C5	C8	C30	C13	C136	C032
C6	C32	C176	C84	C59	

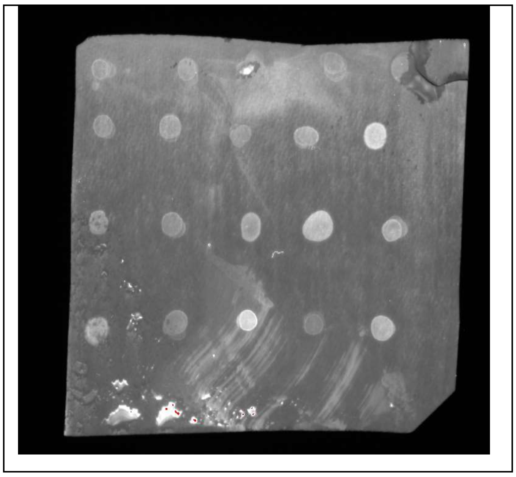

Supplementary Figure S2: Dot blot image of GCH1 in PD CSF

C	PD1209	PD1044	PD1219	PD1039
PD1001	PD1040	PD1216	PD1217	PD0740
PD0816	PD851	PD989	PD1003	PD1004
PD1124	PD1148	PD1167	PD1161	

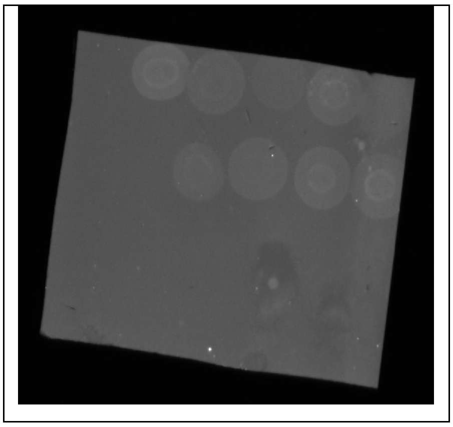

Supplementary Figure S3: Dot blot image of GCH1 control tissue

C	C087	PDC034	PDC035	PDC059
PDC068	PDC084	PDC114	PDC126	

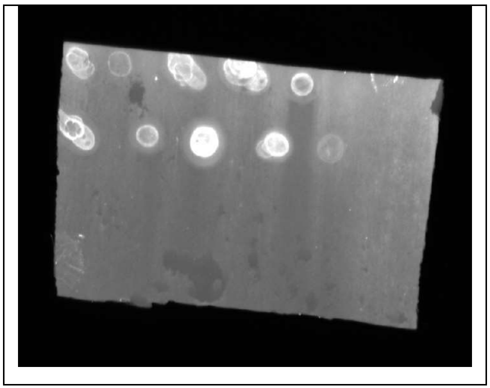

Supplementary Figure S4: Dot blot image of GCH1 PD tissue

C	PD989	PD1003	PD1004	PD1029
PD1040	PD1044	PD1167	PD1209	PD1216

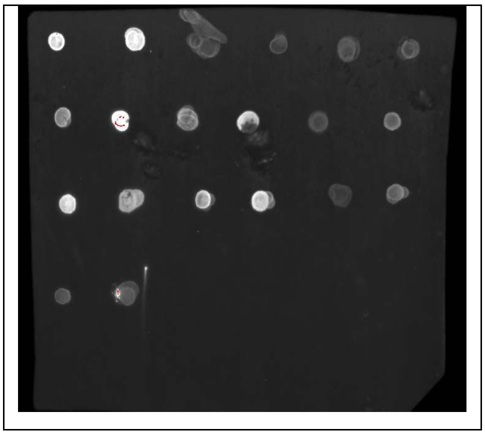

Supplementary Figure S5: Dot blot image of SPR in control CSF

C	PDC114	PDC126	PDC131	PDC68	PDC128
C34	PDC165	C074	C5	C8	C30
C13	C136	C032	C6	C32	C176
C84	C59				

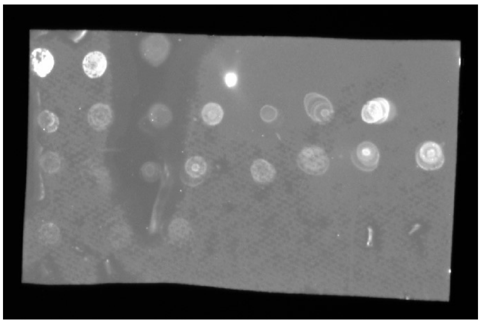

Supplementary Figure S6: Dot blot image of SPR in PD CSF

C	PD1209	PD1044					
PD1219	PD1039	PD1001	PD1040	PD1216	PD1217	PD0740	
PD0816	PD851	PD989	PD1003	PD1004	PD1124	PD1148	PD1167
PD1161							

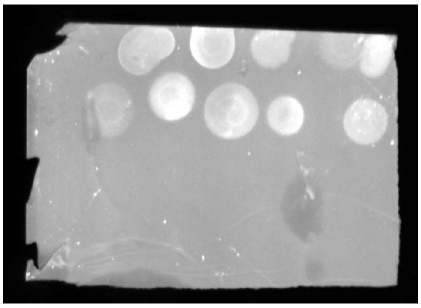

Supplementary Figure S7: Dot blot image of SPR control tissue

C	C087	PDC034	PDC035	PDC059
PDC068	PDC084	PDC114	PDC126	C36

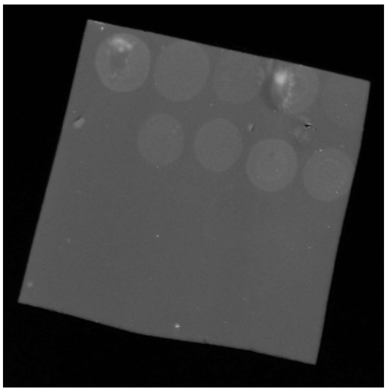

Supplementary Figure S8: Dot blot image of SPR PD tissue

C	PD989	PD1003	PD1004	PD1029
PD1040	PD1044	PD1167	PD1209	PD1216

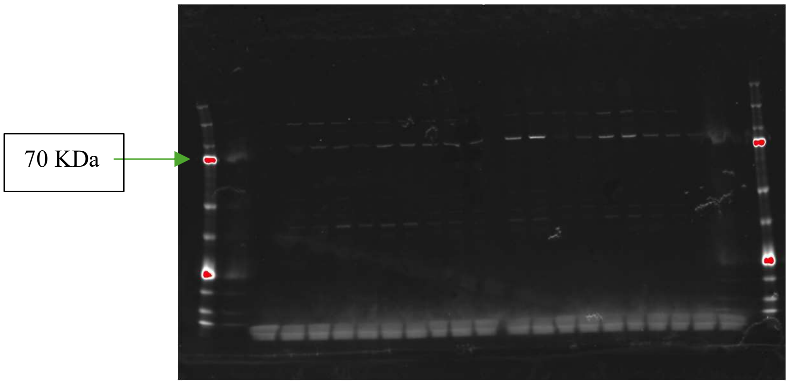

Supplementary Figure S9: Western blot image of TH in control CSF from left to right appearing as mentioned in the table

C	PDC114	PDC126	PDC131	PDC68	PDC128	C34	PDC165	C074	C5
C8	C30	C13	C136	C032	C6	C32	C176	C84	C59

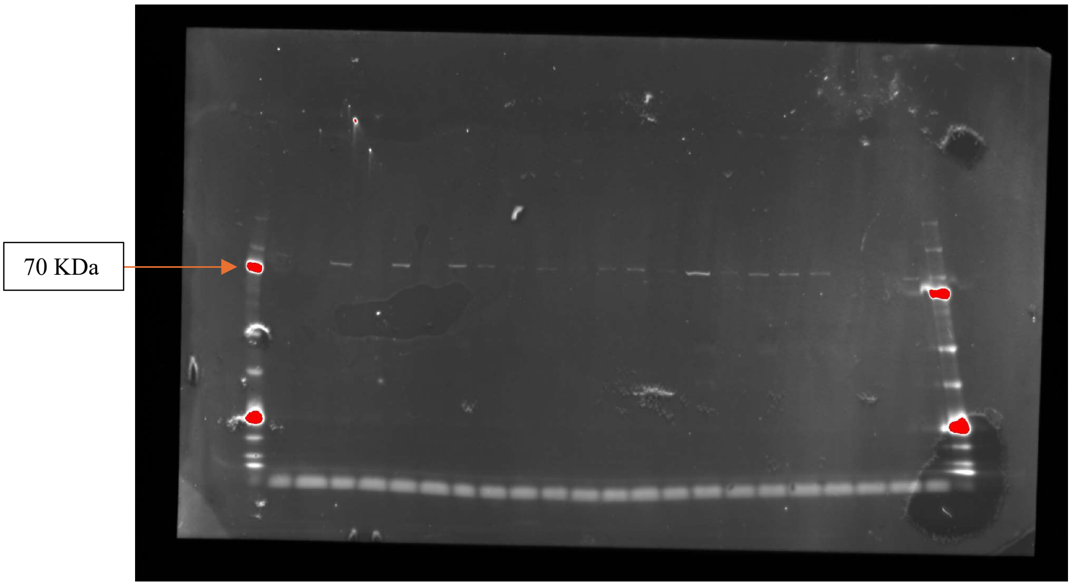

Supplementary Figure S10: Western blot image of TH in PD CSF from left to right appearing as mentioned in the table

C	PD1209	PD1044	PD1219	PD1039	PD1001	PD1040	PD1216	PD1217	PD0740
PD0816	PD851	PD989	PD1003	PD1004	PD1124	PD1148	PD1167	PD1161	

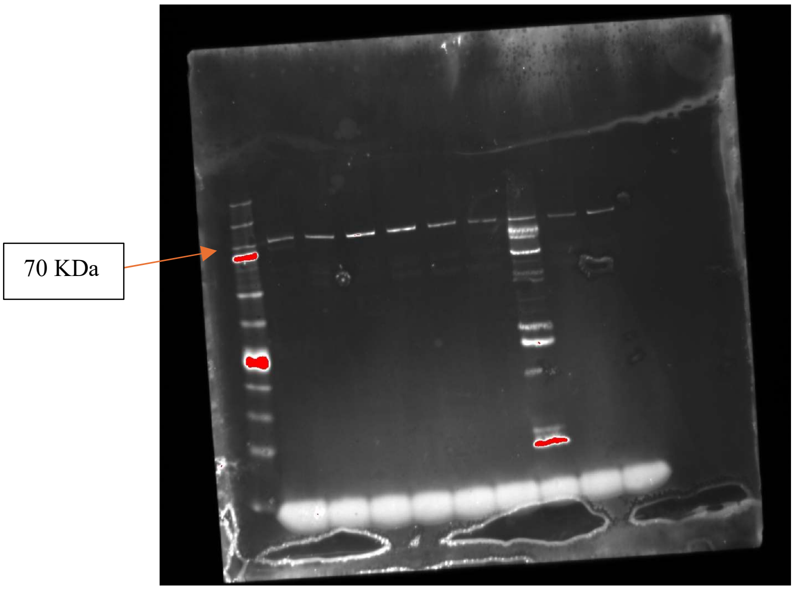

Supplementary Figure S11: Western blot image of TH control tissue from left to right appearing as mentioned in the table

C	C087	PDC034	PDC035	PDC059	PDC068	PDC084	PDC114	PDC126

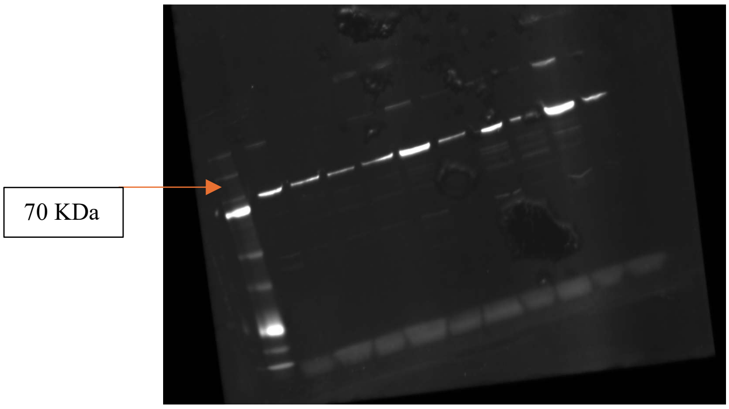

Supplementary Figure S12: Western blot image of TH PD tissue from left to right appearing as mentioned in the table

C	PD989	PD1003	PD1004	PD1029	PD1040	PD1044	PD1167	PD1209	PD1216

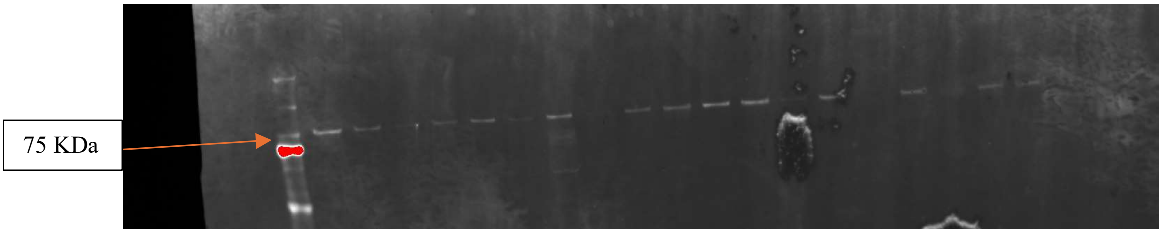

Supplementary Figure S13: Western blot image of MTHFR in control CSF from left to right appearing as mentioned in the table

C	PDC114	PDC126	PDC131	PDC68	PDC128	C34	PDC165	C074	C5

C8	C30	C13	C136	C032	C6	C32	C176	C84	C59

75 KDa

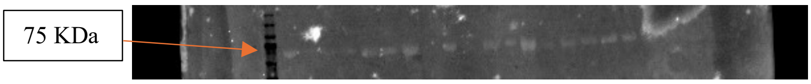

Supplementary Figure S14: Western blot image of MTHFR in PD CSF from left to right appearing as mentioned in the table

C	PD1209	PD1044	PD1219	PD1039	PD1001	PD1040	PD1216	PD1217	PD0740
PD0816	PD851	PD989	PD1003	PD1004	PD1124	PD1148	PD1167	PD1161	

100 KDa

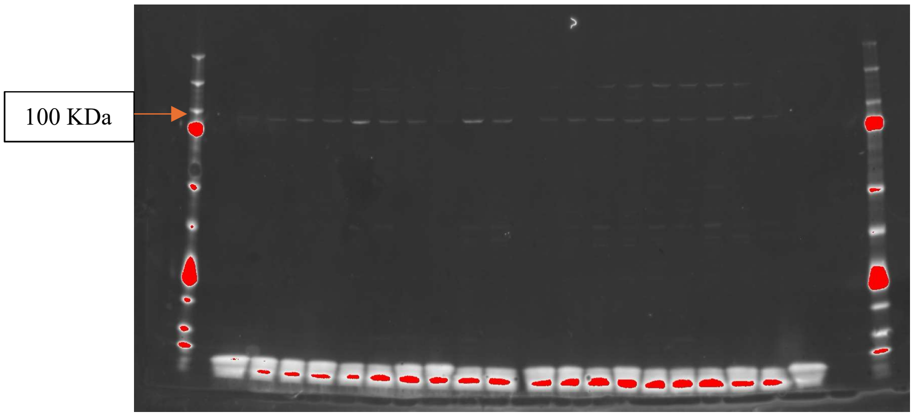

Supplementary Figure S15: Western blot image of MTHFD1 in control CSF from left to right appearing as mentioned in the table

C	PDC114	PDC126	PDC131	PDC68	PDC128	C34	PDC165	C074	C5
C8	C30	C13	C136	C032	C6	C32	C176	C84	C59

100 KDa

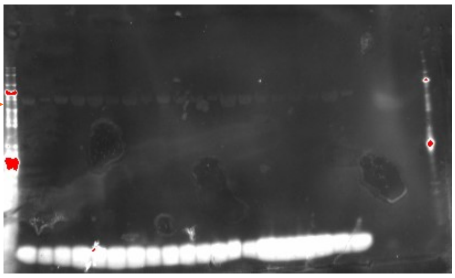

Supplementary Figure S16: Western blot image of MTHFD1 in PD CSF from left to right appearing as mentioned in the table

C	PD1209	PD1044	PD1219	PD1039	PD1001	PD1040	PD1216	PD1217	PD0740
PD0816	PD851	PD989	PD1003	PD1004	PD1124	PD1148	PD1167	PD1161	

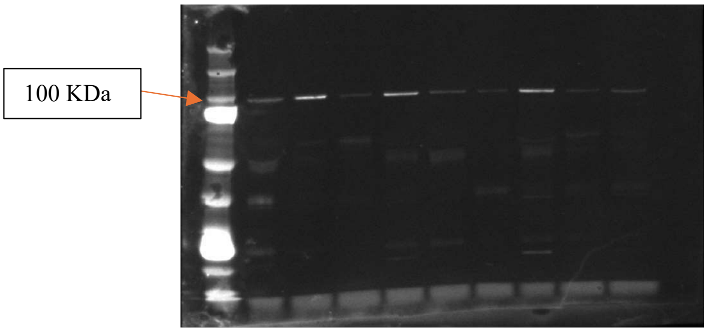

Supplementary Figure S17: Western blot image of MTHFD1 control tissue from left to right appearing as mentioned in the table

C	C087	PDC034	PDC035	PDC059	PDC068	PDC084	PDC114	PDC126

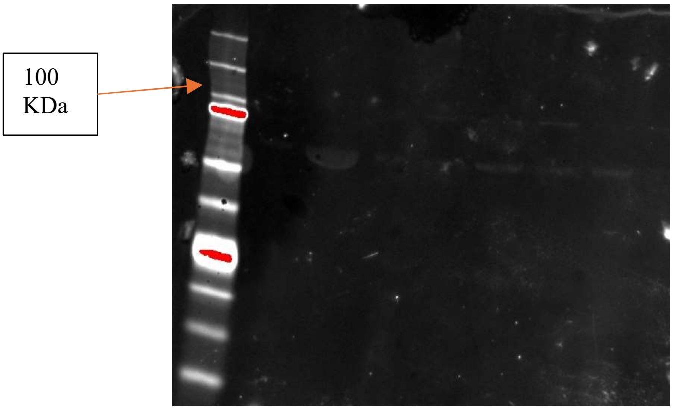

Supplementary Figure S18: Western blot image of MTHFD1 PD tissue from left to right appearing as mentioned in the table

C	PD989	PD1003	PD1004	PD1029	PD1040	PD1044	PD1167	PD1209	PD1216

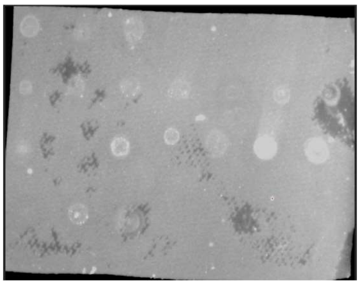

Supplementary Figure S19: Dot blot image of PTPS in control CSF from left to right appearing as mentioned in the table

C	PDC114	PDC126				
PDC131	PDC68	PDC128	C34	PDC165	C074	C5
C8	C30	C13	C136	C032	C6	C32
C176	C84	C59				

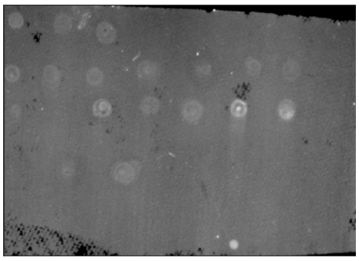

Supplementary Figure S20: Dot blot image of PTPS in PD CSF from left to right appearing as mentioned in the table

C	PD1209	PD1044				
PD1219	PD1039	PD1001	PD1040	PD1216	PD1217	PD0740
PD0816	PD851	PD989	PD1003	PD1004	PD1124	
PD1148	PD1167	PD1161				

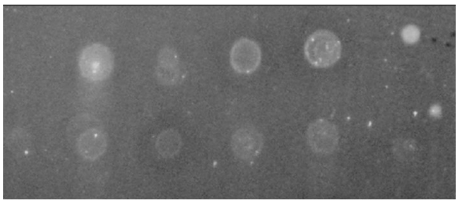

Supplementary Figure S21: Dot blot image of PTPS control tissue from left to right appearing as mentioned in the table

C	C087	PDC034	PDC035	PDC059
PDC068	PDC084	PDC114	PDC126	

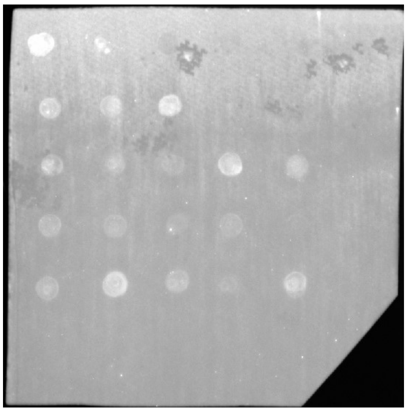

Supplementary Figure S22: Dot blot image of PTPS in PD Tissue from left to right appearing as mentioned in the table

C	PD1209	PD1044		
PD1219	PD1039	PD1001		
PD1040	PD1216	PD1217	PD0740	PD0816
PD851	PD989	PD1003	PD1004	
PD1124	PD1148	PD1167	PD1161	

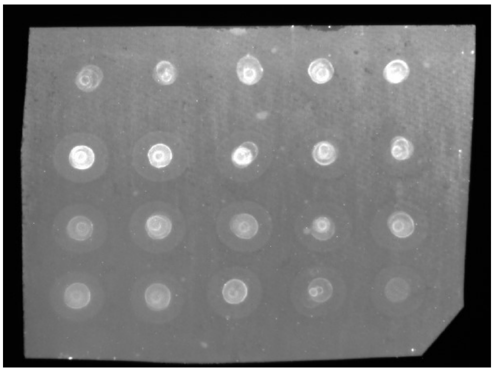

Supplementary Figure S23: Dot blot image of DHFR in control CSF from left to right appearing as mentioned in the table

C	PDC114	PDC126	PDC131	PDC68
PDC128	C34	PDC165	C074	C5
C8	C30	C13	C136	C032
C6	C32	C176	C84	C59

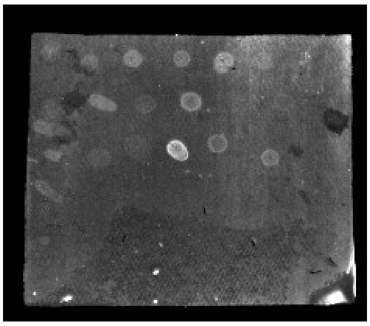

Supplementary Figure S24: Dot blot image of DHFR in PD CSF from left to right appearing as mentioned in the table

C	PD1209	PD1044	PD1219	PD1039	PD1001	PD1040	PD1216	PD1217	PD0740
PD0816	PD851	PD989	PD1003	PD1004	PD1124	PD1148	PD1167	PD1161	

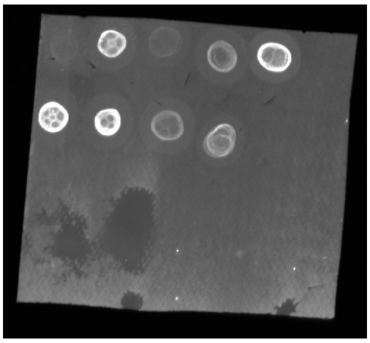

Supplementary Figure S25: Dot blot image of DHFR control tissue from left to right appearing as mentioned in the table

C	C087	PDC034	PDC035	PDC059
PDC068	PDC084	PDC114	PDC126	

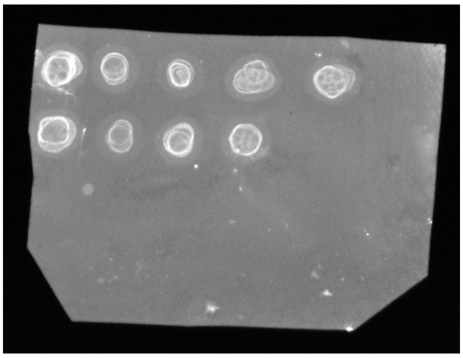

Supplementary Figure S26: Dot blot image of DHFR PD tissue from left to right appearing as mentioned in the table

C	PD989	PD1003	PD1004	PD1029
PD1040	PD1044	PD1167	PD1209	PD1216
